# Supplementary material for: Engineered bacterial outer membrane vesicles encapsulating oncolytic adenoviruses enhance the efficacy of cancer virotherapy by augmenting tumor cell autophagy
Source: Nat Commun. 2023 May 22;14:2933. doi: 10.1038/s41467-023-38679-z (PMC10203215; doi:10.1038/s41467-023-38679-z)
Supplement: Supplementary file 1 — Supplementary Information [file 41467_2023_38679_MOESM1_ESM.pdf]

# Supplementary Information

# Engineered bacterial outer membrane vesicles encapsulating oncolytic adenoviruses enhance the efficacy of cancer virotherapy by augmenting tumor cell autophagy

**Ban et al.**

**Supplementary Figures**

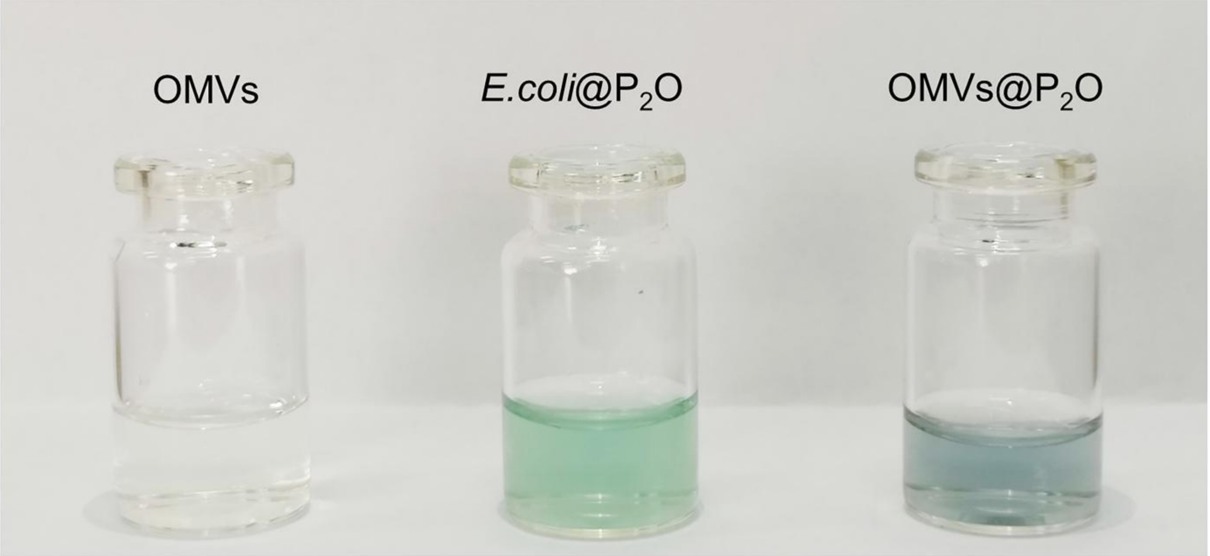


**Supplementary Figure 1.** Qualitative analysis of $P_2O$ expression in OMVs, *E. coli*@$P_2O$ and OMVs@$P_2O$. This experiment was repeated three times independently with similar results.

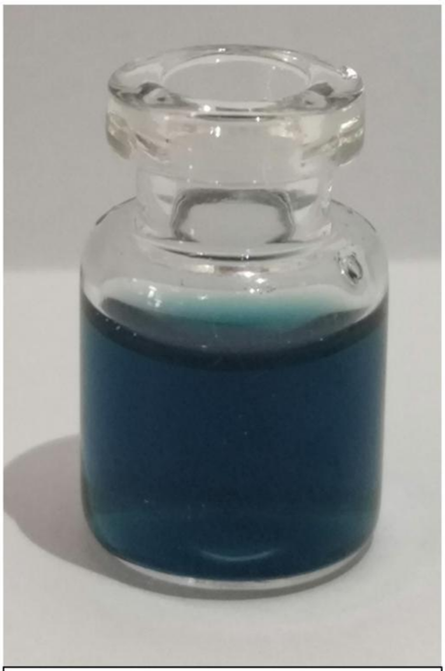

OMVs@$P_2O$-Ads

**Supplementary Figure 2.** Qualitative analysis of $P_2O$ expression in OMVs@$P_2O$-Ads. This experiment was repeated three times independently with similar results.

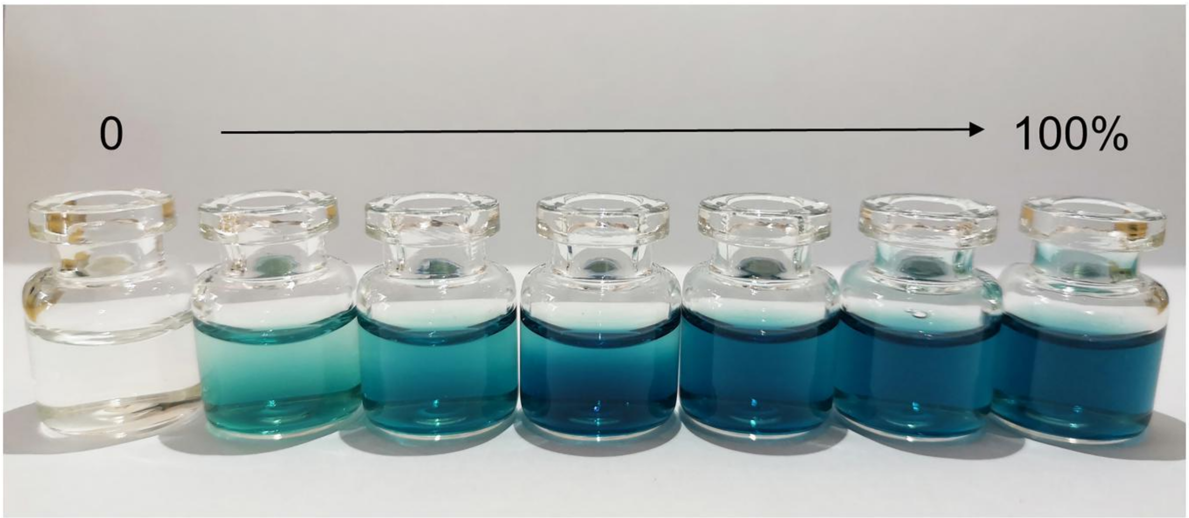


**Supplementary Figure 3.** Qualitative analysis of different concentration $P_2O$ expression in OMVs@$P_2O$. This experiment was repeated three times independently with similar results.

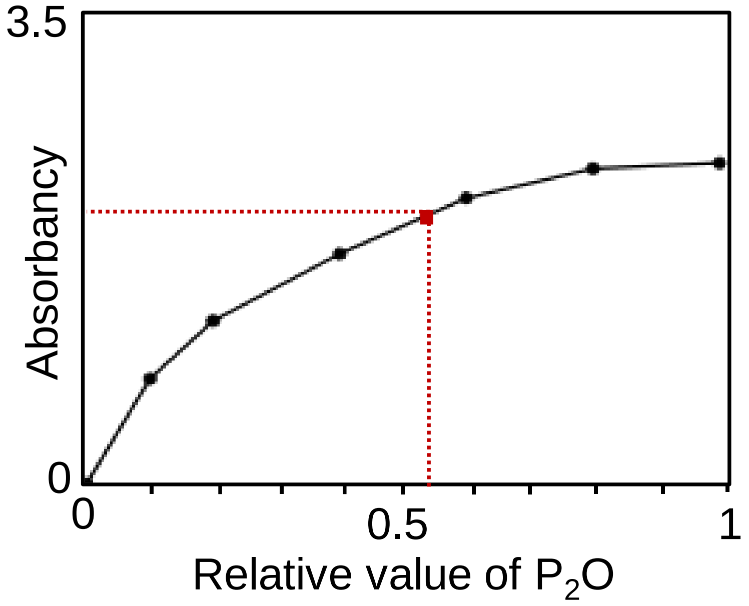


**Supplementary Figure 4.** The function curve illustrated the relationship between the absorbance and $P_2O$ with different concentrations. The red point revealed the relative $P_2O$ concentration within the microbial nanocomposite. Source data are provided as a Source Data file. This experiment was repeated three times independently with similar results.

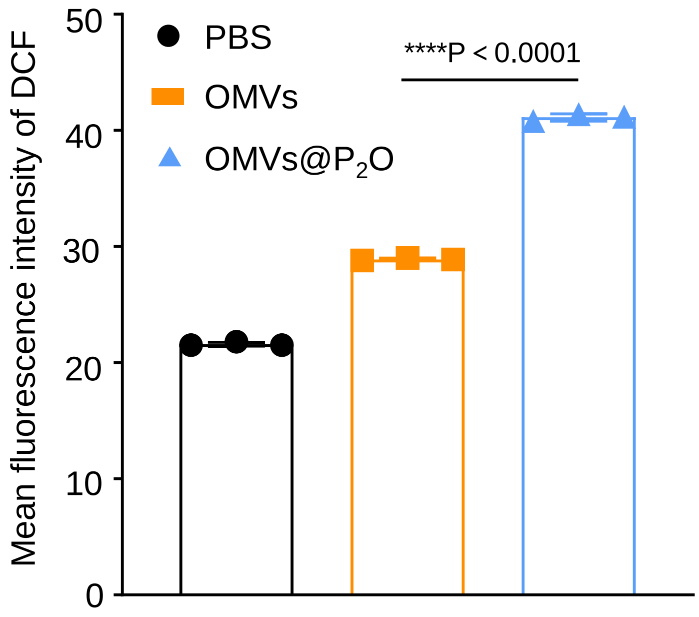


**Supplementary Figure 5.** Quantitation of mean fluorescence intensity of DCF in PBS, OMVs and OMVs@$P_2O$ for ROS level assessment in cells. Data are presented as mean ± SD (n = 3 independent experiments). ****$P < 0.0001$ by two-tailed Student's t-test. Source data are provided as a Source Data file.

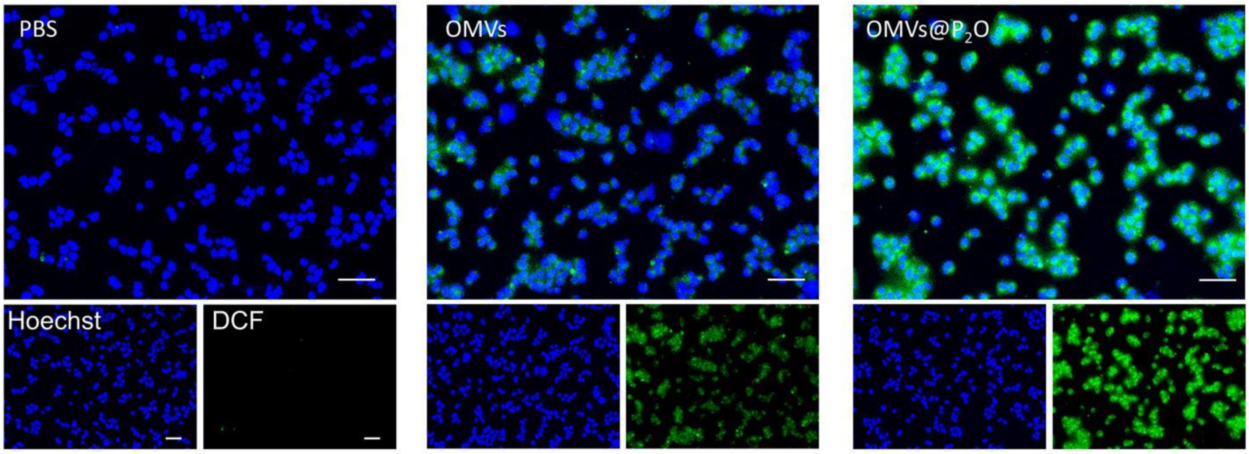


**Supplementary Figure 6.** Immunofluorescence images of ROS expression in tumor cells in vitro. The cells were stained with DAPI (blue), and ROS were stained with DCF (green). Scale bars=100 μm. This experiment was repeated three times independently with similar results.

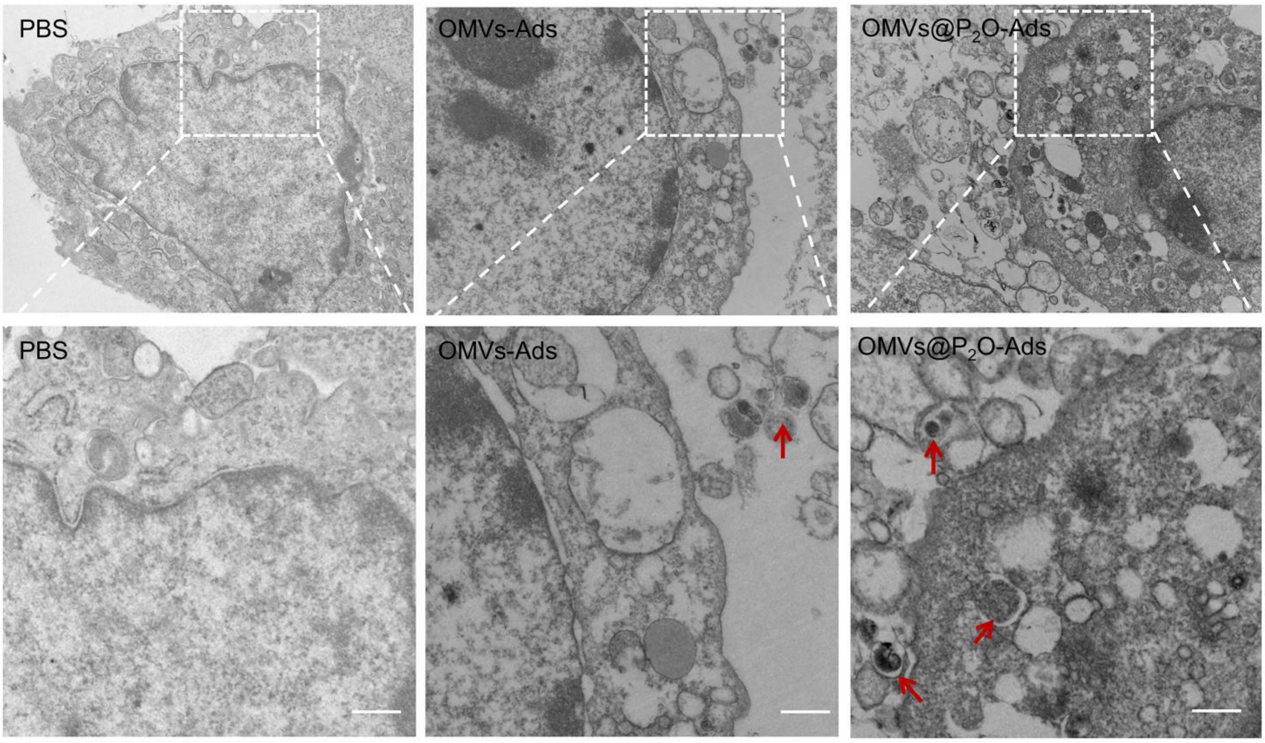


33

34 **Supplementary Figure 7.** The equal-scaling amplifying TEM images of autophagosomes, scale

35 bar=500 nm. This experiment was repeated three times independently with similar results.

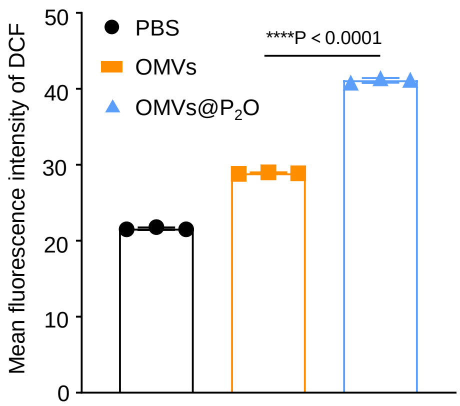


**Supplementary Figure 8.** The LC3-II/LC3-I ratio in vitro. Data are presented as mean ± SD (n = 3 independent experiments). ***P* < 0.01 by two-tailed Student's t-test. Source data are provided as a Source Data file.

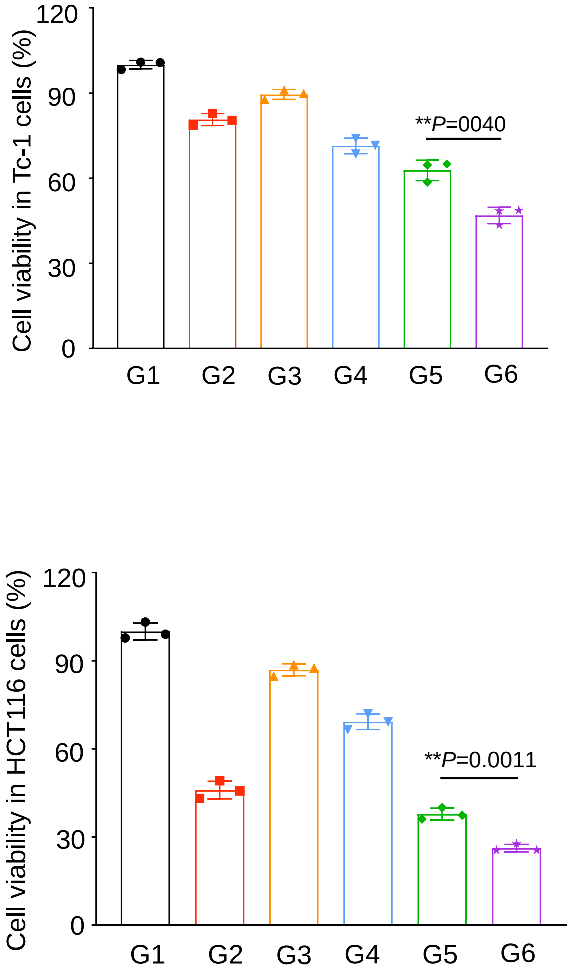


**Supplementary Figure 9.** Cytotoxicity of different formulations in TC-1 cells and HCT116 cells by MTT assay. Data are presented as mean ± SD (n = 3 independent experiments). $^{**}P < 0.01$ by two-tailed Student's t-test. Source data are provided as a Source Data file. (G1: PBS, G2: Ads, G3: OMVs, G4: OMVs@$P_2O$, G5: OMVs-Ads, G6: OMVs@$P_2O$-Ads).

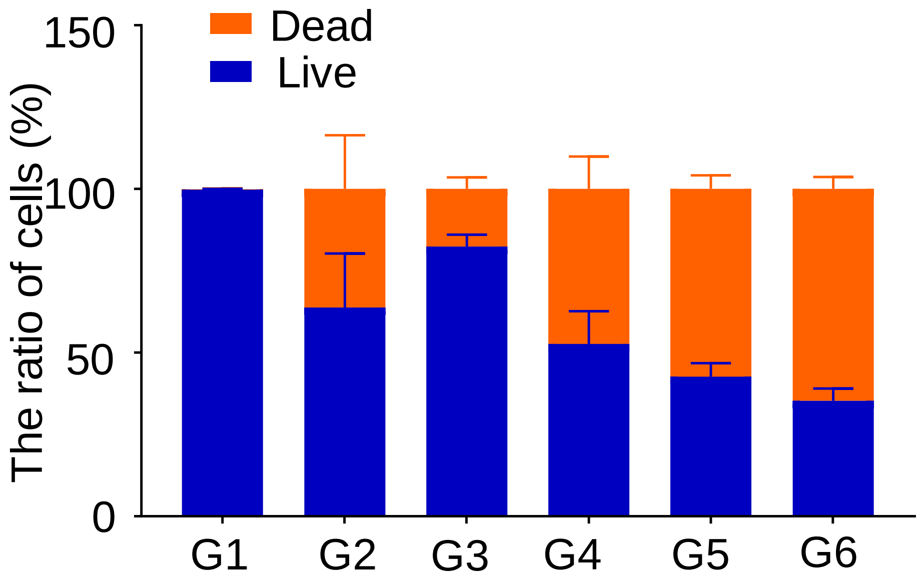


**Supplementary Figure 10.** The statistical analysis result of live/dead cellular staining. Data are presented as mean ± SD. Source data are provided as a Source Data file. (G1: PBS, G2: Ads, G3: OMVs, G4: OMVs@$P_2O$, G5: OMVs-Ads, G6: OMVs@$P_2O$-Ads). This experiment was repeated three times independently with similar results.

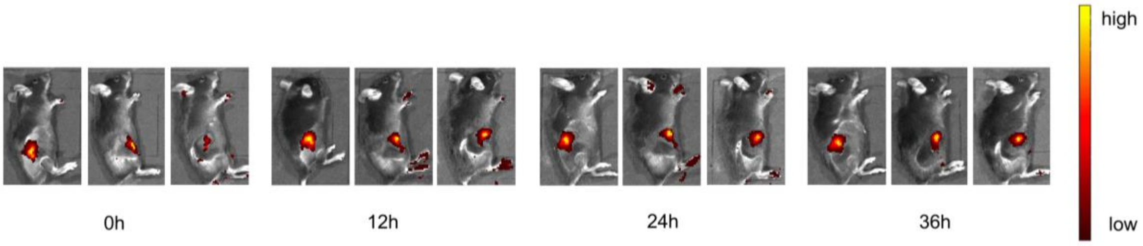


**Supplementary Figure 11**. In vivo DIR fluorescent imaging of the nanocomposite in TC-1-hCD46 xenograft tumor-bearing mice by IVIS (n = 4 mice).

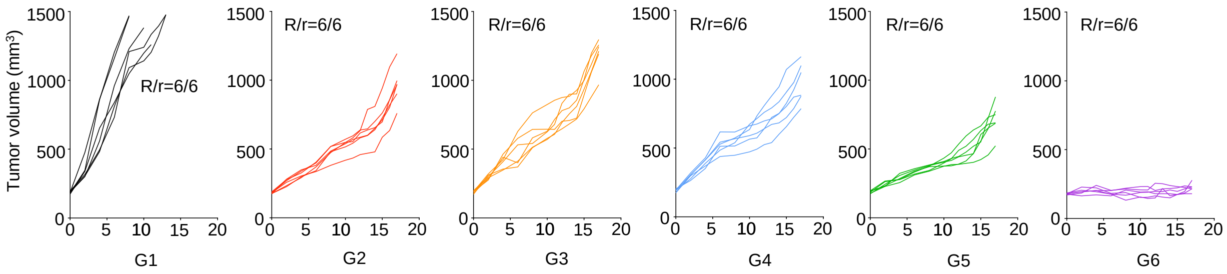


**Supplementary Figure 12.** Individual tumor growth kinetics in different groups. (n = 6 mice). Source data are provided as a Source Data file. (G1: PBS, G2: Ads, G3: OMVs, G4: OMVs@$P_2O$, G5: OMVs-Ads, G6: OMVs@$P_2O$-Ads).

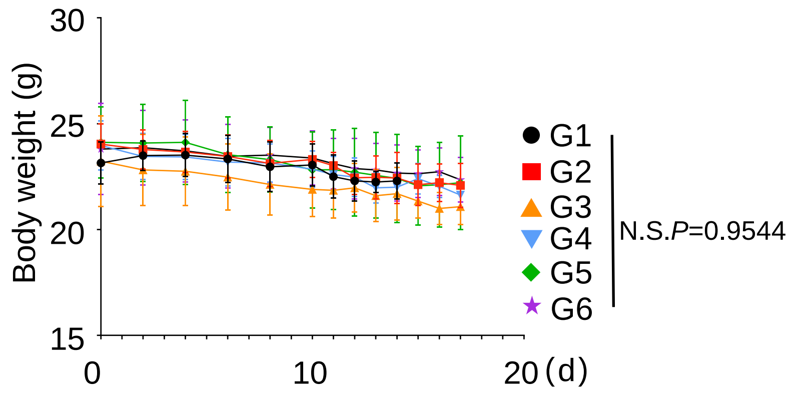


**Supplementary Figure 13.** Body weight changes of TC-1-bearing mice after intratumoral administration of different formulations. Data are presented as mean ± SD (n = 6 mice). N.S. (No Significance) $P > 0.05$ by two-tailed Student's t-test. Source data are provided as a Source Data file. (G1: PBS, G2: Ads, G3: OMVs, G4: OMVs@$P_2O$, G5: OMVs-Ads, G6: OMVs@$P_2O$-Ads).

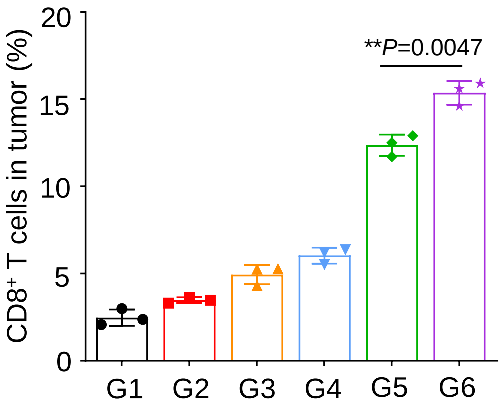


**Supplementary Figure 14.** The infiltration of $CD8^{+}$ T cells in tumor of mice treated with different agents on the 18th day. Data are presented as mean ± SD (n = 3 mice). $^{**}P < 0.01$ by two-tailed Student's t-test. Source data are provided as a Source Data file. (G1: PBS, G2: Ads, G3: OMVs, G4: OMVs@$P_2$O, G5: OMVs-Ads, G6: OMVs@$P_2$O-Ads).

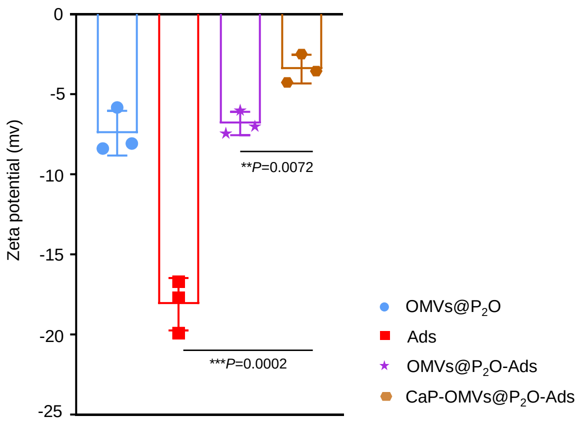


**Supplementary Figure 15.** Zeta-potential of OMVs@$P_2O$, Ads, OMVs@$P_2O$-Ads and CaP-OMVs@$P_2O$-Ads. Data are presented as mean ± SD (n = 3 independent experiments). $^{**}P < 0.01$ and $^{***}P < 0.001$ by two-tailed Student's t-test. Source data are provided as a Source Data file.

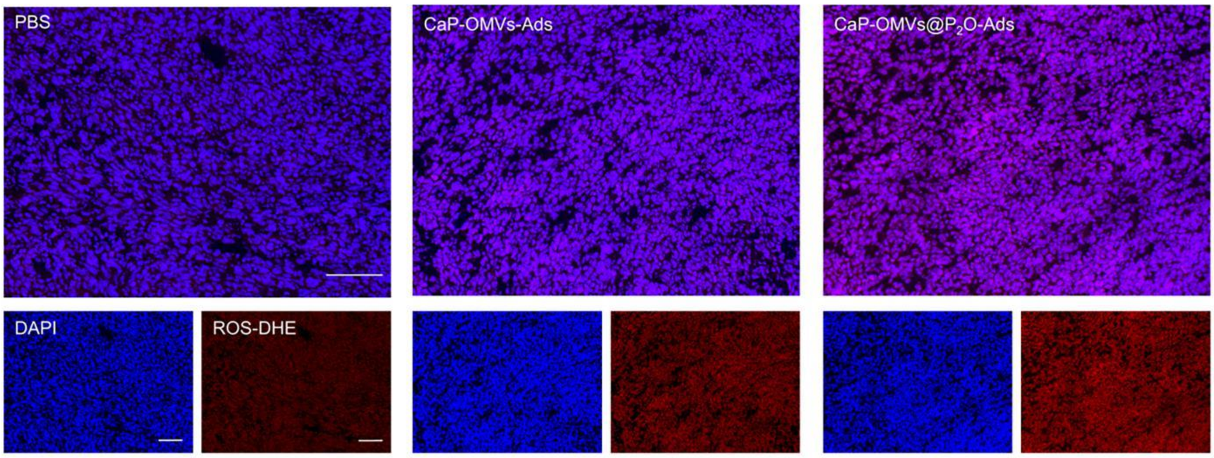


**Supplementary Figure 16.** Immunofluorescence images of ROS in tumor tissues. Blue represents DAPI-stained tumor cells and red represents DHE. Scale bar=100 μm. This experiment was repeated three mice with similar results.

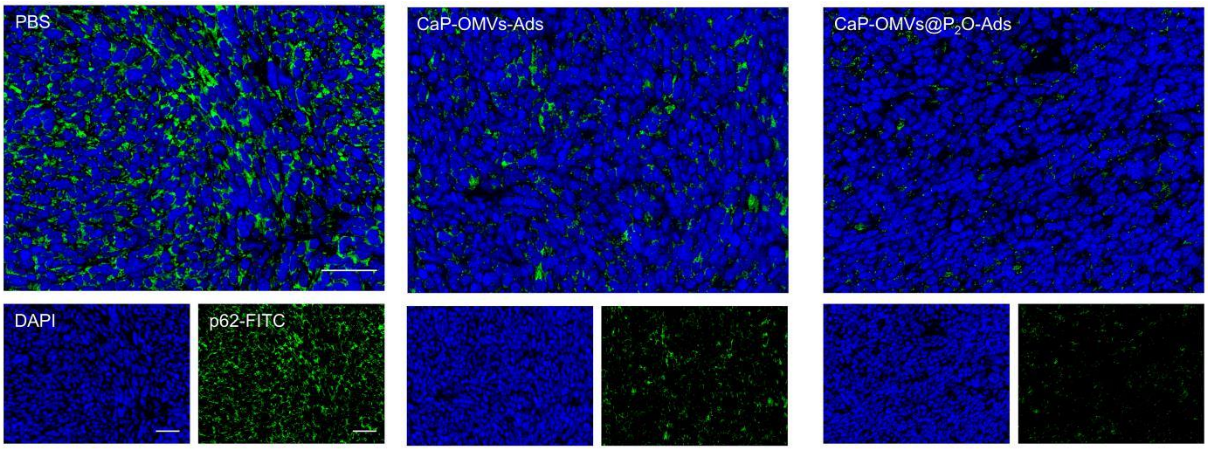


**Supplementary Figure 17.** Immunofluorescence images of p62 autophagic proteins in tumor tissues. Blue represents DAPI-stained tumor cells and the green represents FITC-stained p62 autophagic protein. Scale bar=50 μm. This experiment was repeated three mice with similar results.

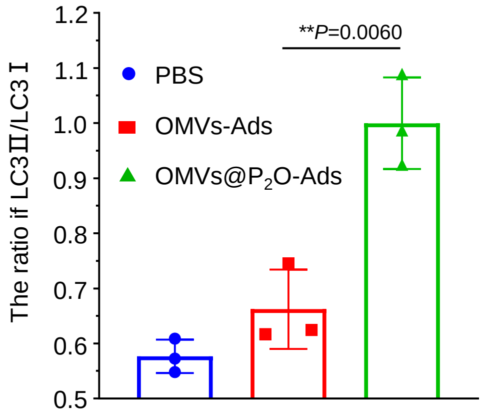


**Supplementary Figure 18.** The LC3-II/LC3-I ratio in vivo. Data are presented as mean ± SD (n = 3 mice). *$P$ < 0.01 by two-tailed Student's t-test. Source data are provided as a Source Data file.

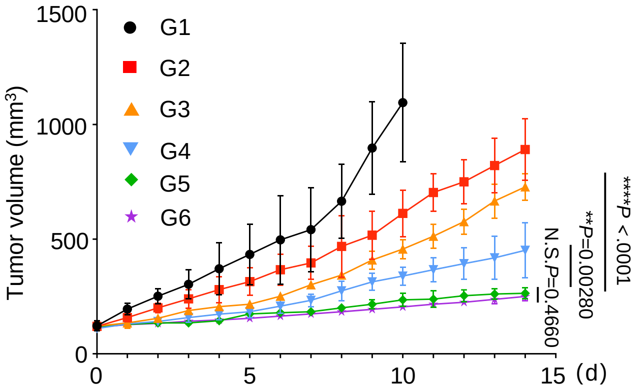


**Supplementary Figure 19.** The tumor of TC-1-bearing mice model volume change for TC-1 xenograft tumor model during different treatments. Data are presented as mean ± SD (n = 6 mice). N.S. (No Significance) $P$ > 0.05, **$P$ < 0.01 and ****$P$ < 0.0001 by two-tailed Student's t-test. Source data are provided as a Source Data file. (G1: PBS, G2: OMVs@$P_2O$-Ads, G3: CaP-OMVs-Ads, G4: Intra-Ads, G5: CaP-OMVs@$P_2O$-Ads, G6: Intra-Ads high does).

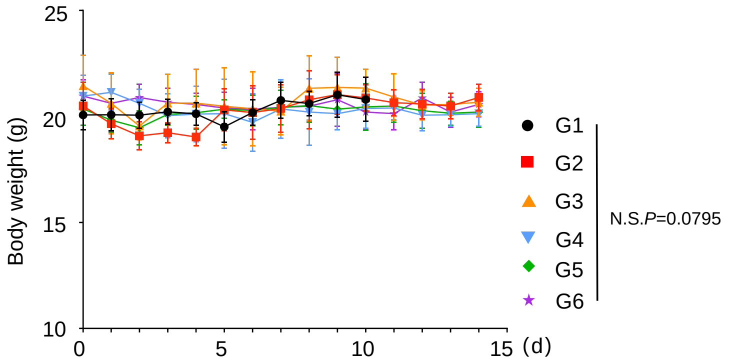


**Supplementary Figure 20.** Body weight changes of TC-1-bearing mice after administration of different formulations. Data are presented as mean ± SD (n = 6 mice). N.S. (No Significance) $P > 0.05$ by two-tailed Student's t-test. Source data are provided as a Source Data file. (G1: PBS, G2: OMVs@$P_2$O-Ads, G3: CaP-OMVs-Ads, G4: Intra-Ads, G5: CaP-OMVs@$P_2$O-Ads, G6: Intra-Ads high does).

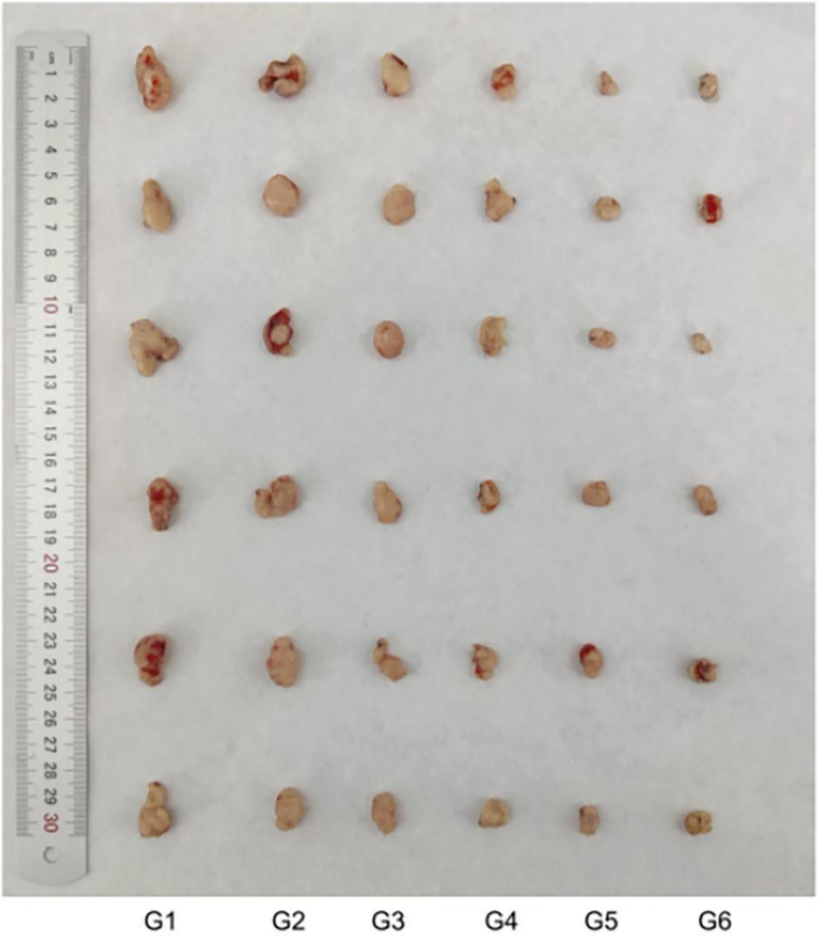


**Supplementary Figure 21.** Images of representative tumors after different treatments at 15 days (n=6). (G1: PBS, G2: OMVs@$P_2$O-Ads, G3: CaP-OMVs-Ads, G4: Intra-Ads, G5: CaP-OMVs@$P_2$O-Ads, G6: Intra-Ads high does).

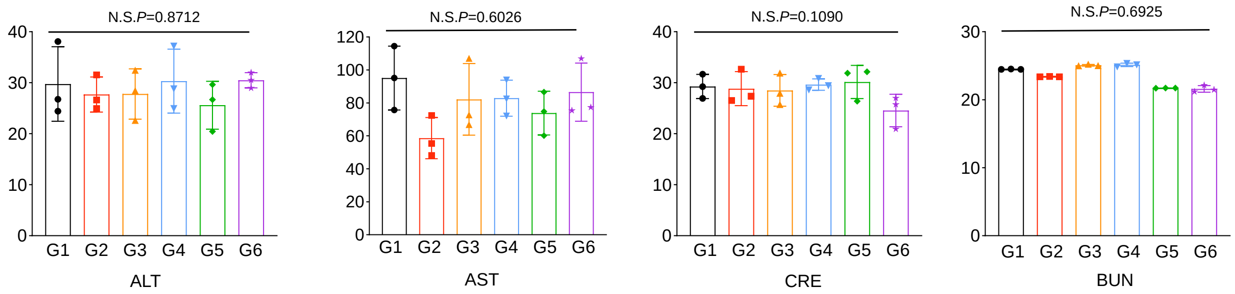


**Supplementary Figure 22.** Hepatorenal function indexes including aspartate aminotransferase (AST), alanine aminotransferase (ALT) levels, creatinine (CRE) and blood urea nitrogen (BUN) were measured. Data are presented as mean ± SD (n = 3 mice). N.S. (No Significance) $P > 0.05$ by two-tailed Student's t-test. Source data are provided as a Source Data file. (G1: PBS, G2: OMVs@$P_2$O-Ads, G3: CaP-OMVs-Ads, G4: Intra-Ads, G5: CaP-OMVs@$P_2$O-Ads, G6: Intra-Ads high does).

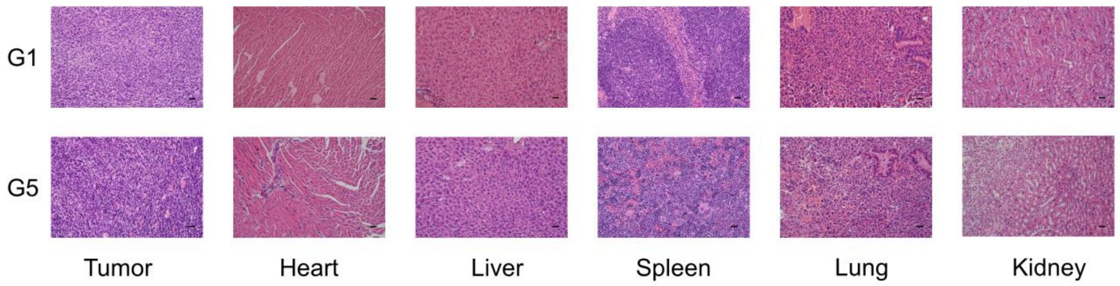


**Supplementary Figure 23.** H&E staining images for major organs slices after different treatments. Scale bar=100 μm. This experiment was repeated three mice with similar results.

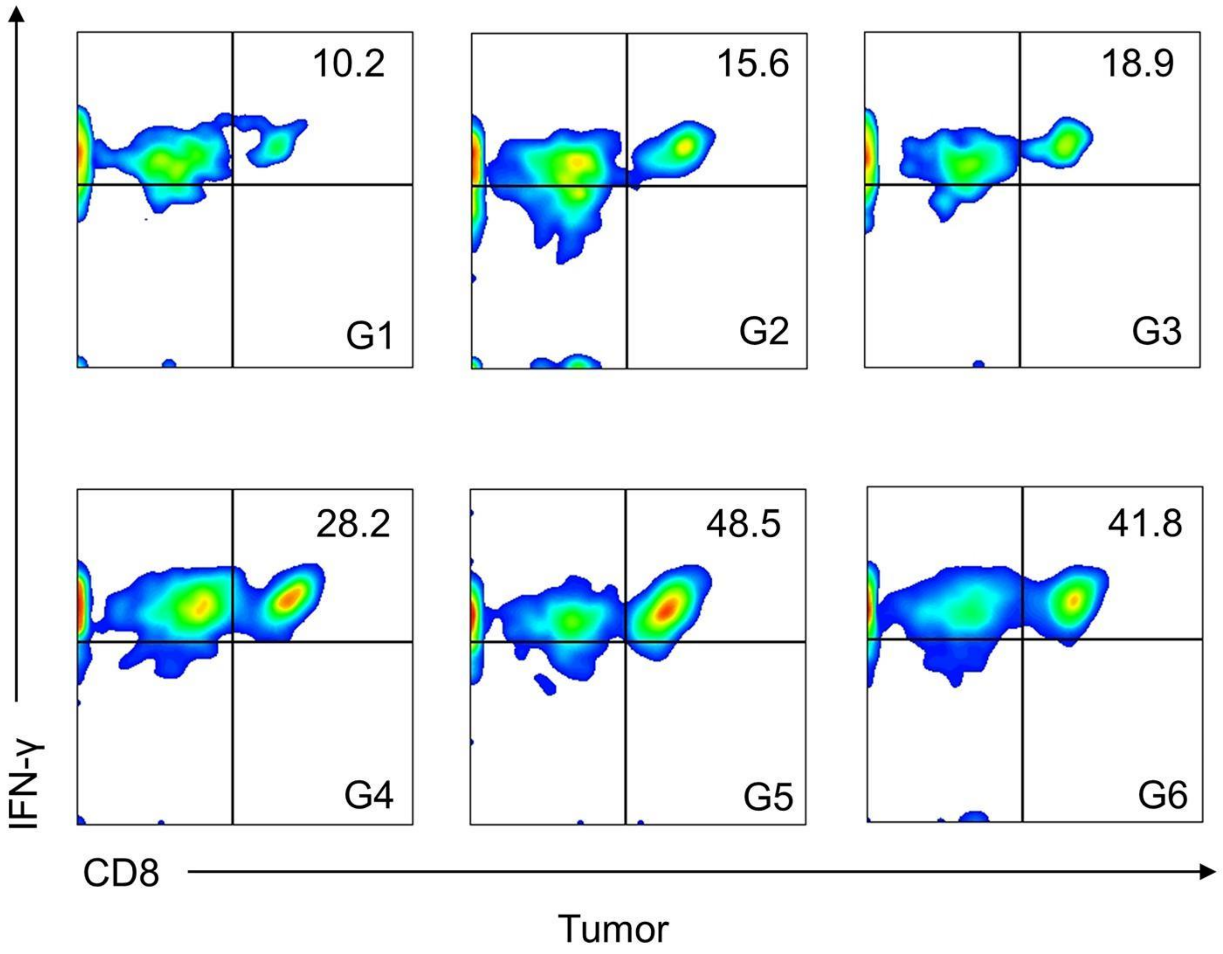


**Supplementary Figure 24.** Representative flow cytometric evolution images of IFN-$\gamma^+$CD8$^+$ T cells (CD45$^+$CD3$^+$CD8$^+$IFN-$\gamma^+$) in tumor. (G1: PBS, G2: OMVs@$P_2$O-Ads, G3: CaP-OMVs-Ads, G4: Intra-Ads, G5: CaP-OMVs@$P_2$O-Ads, G6: Intra-Ads high does)

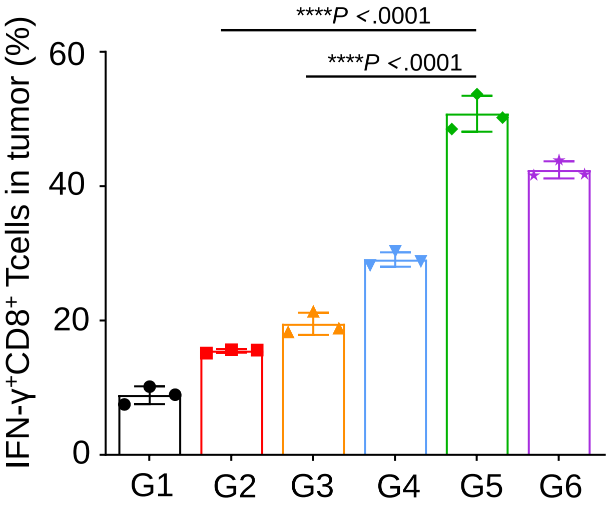


**Supplementary Figure 25.** Relative quantification of IFN-$\gamma^+$CD8$^+$ T cells (CD45$^+$CD3$^+$CD8$^+$IFN-$\gamma^+$) in tumor. Data are presented as mean ± SD (n = 3 mice). ****$P$ < 0.0001 by two-tailed Student's t-test. Source data are provided as a Source Data file. (G1: PBS, G2: OMVs@$P_2$O-Ads, G3: CaP-OMVs-Ads, G4: Intra-Ads, G5: CaP-OMVs@$P_2$O-Ads, G6: Intra-Ads high does)

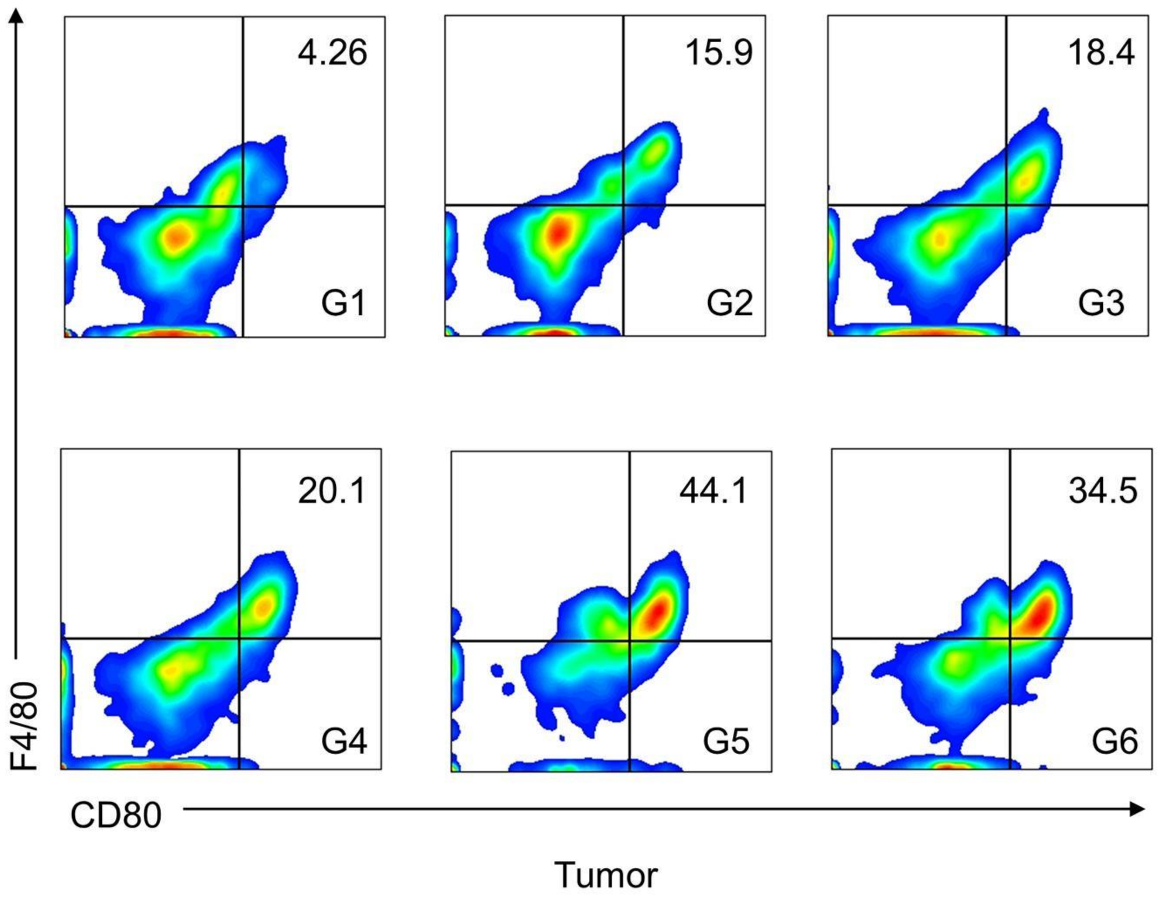


**Supplementary Figure 26.** Representative flow cytometric evolution images of M1-like macrophages (CD45$^{+}$F4/80$^{+}$CD80$^{+}$) in tumor (n=3). (G1: PBS, G2: OMVs@$P_2O$-Ads, G3: CaP-OMVs-Ads, G4: Intra-Ads, G5: CaP-OMVs@$P_2O$-Ads, G6: Intra-Ads high does)

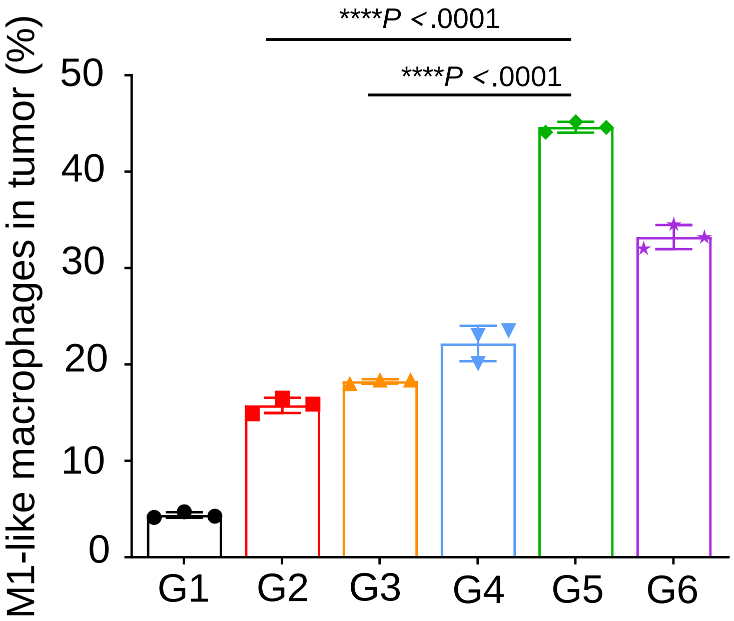


**Supplementary Figure 27.** Relative quantification of M1-like macrophages ($CD45^+F4/80^+CD80^+$) in tumor. Data are presented as mean ± SD (n = 3 mice). ****$P < 0.0001$ by two-tailed Student's t-test. Source data are provided as a Source Data file. (G1: PBS, G2: OMVs@$P_2$O-Ads, G3: CaP-OMVs-Ads, G4: Intra-Ads, G5: CaP-OMVs@$P_2$O-Ads, G6: Intra-Ads high does)

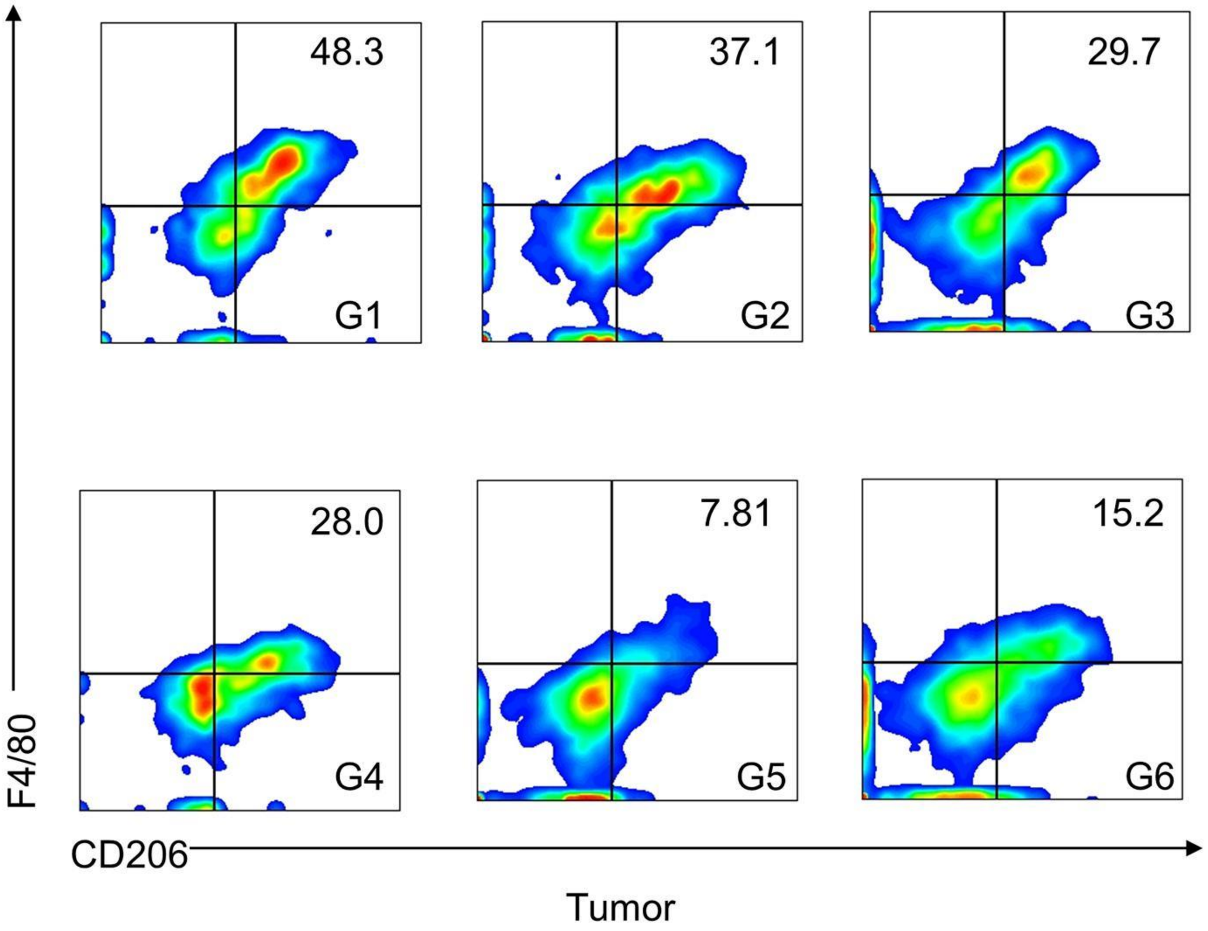


**Supplementary Figure 28.** Representative flow cytometric evolution images of M2-like macrophages ($CD45^{+}F4/80^{+}CD206^{+}$) in tumor (n=3). (G1: PBS, G2: OMVs@$P_2$O-Ads, G3: CaP-OMVs-Ads, G4: Intra-Ads, G5: CaP-OMVs@$P_2$O-Ads, G6: Intra-Ads high does)

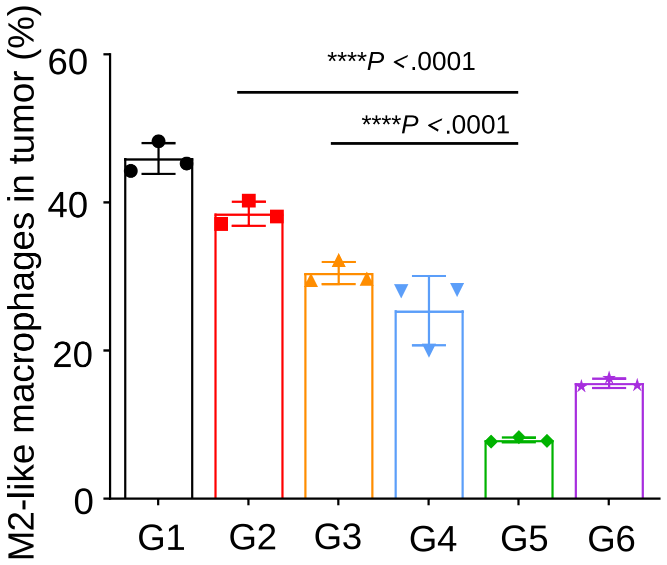


**Supplementary Figure 29.** Relative quantification of M2-like macrophages ($CD45^+F4/80^+CD206^+$) in tumor. Data are presented as mean ± SD (n = 3 mice). ****$P$ < 0.0001 by two-tailed Student's t-test. Source data are provided as a Source Data file. (G1: PBS, G2: OMVs@$P_2O$-Ads, G3: CaP-OMVs-Ads, G4: Intra-Ads, G5: CaP-OMVs@$P_2O$-Ads, G6: Intra-Ads high does)

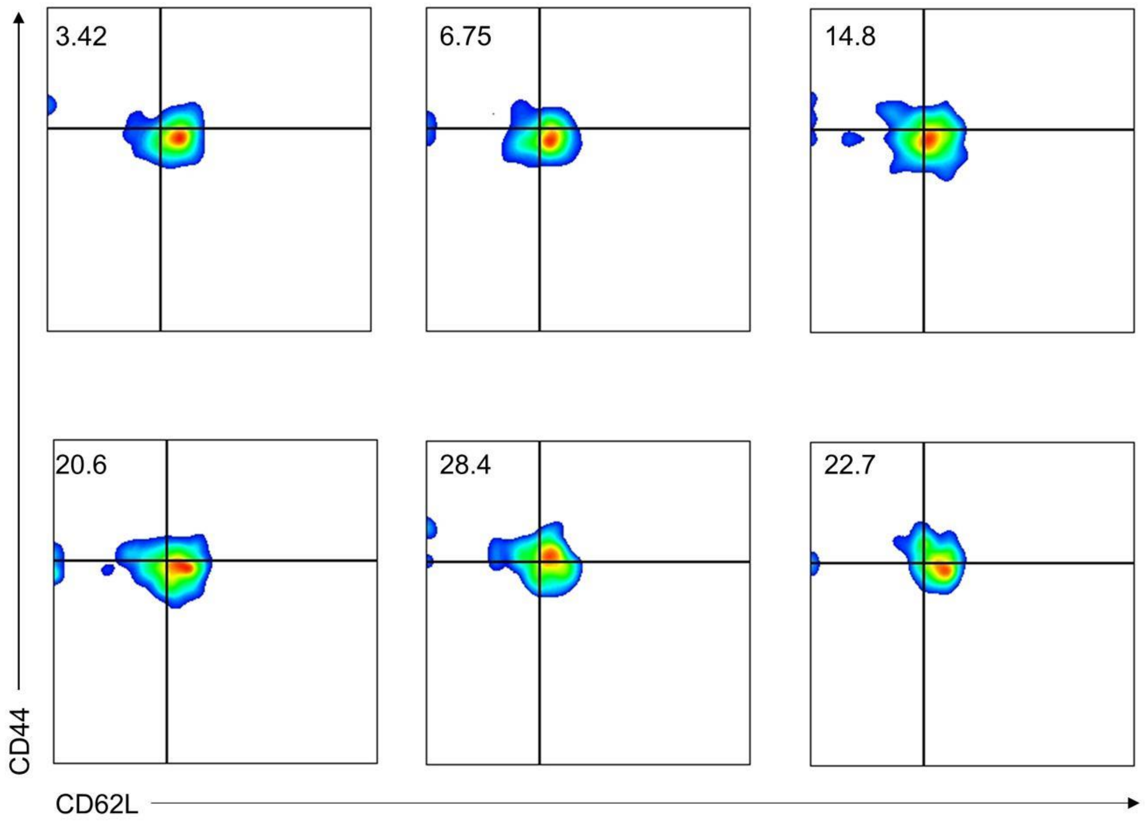


**Supplementary Figure 30.** The gating strategy of effector memory T cells ($CD3^+$ $CD8^+$ $CD62L^-$ $CD44^+$) in spleen. (G1: PBS, G2: OMVs@$P_2$O-Ads, G3: CaP-OMVs-Ads, G4: Intra-Ads, G5: CaP-OMVs@$P_2$O-Ads, G6: Intra-Ads high does).

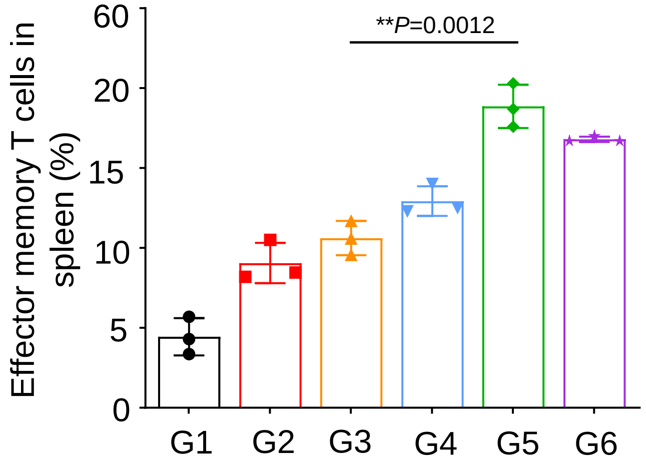


**Supplementary Figure 31.** Relative quantification of effector memory T cells ($CD3^+CD8^+CD62L^-CD44^+$) in spleen. Data are presented as mean ± SD (n = 3 mice). $^{**}P < 0.01$ by two-tailed Student's t-test. Source data are provided as a Source Data file. (G1: PBS, G2: OMVs@$P_2O$-Ads, G3: CaP-OMVs-Ads, G4: Intra-Ads, G5: CaP-OMVs@$P_2O$-Ads, G6: Intra-Ads high does)

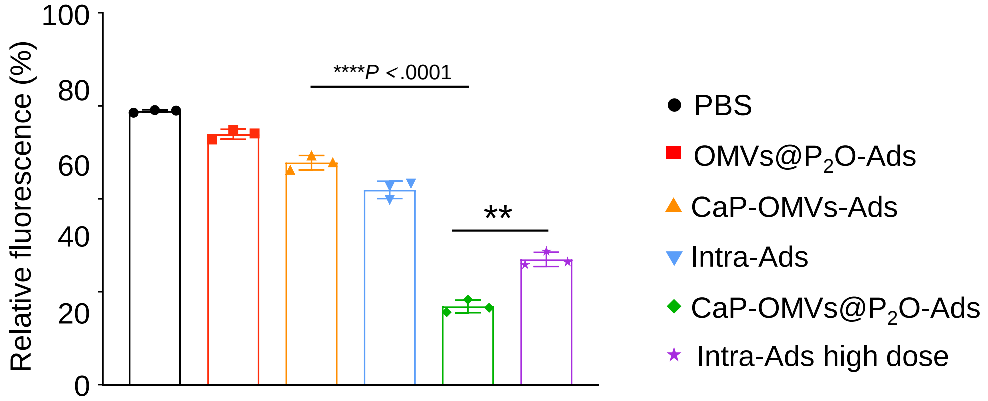


**Supplementary Figure 32.** The experimental result of the co-culture assay. Data are presented as mean ± SD (n = 3 independent experiments). $^{**}P < 0.01$, $^{****}P < 0.0001$ by two-tailed Student's t-test. Source data are provided as a Source Data file. (It's worth noting here that PBS represents T cells extracted from mice in the PBS group, and other groups as above.)

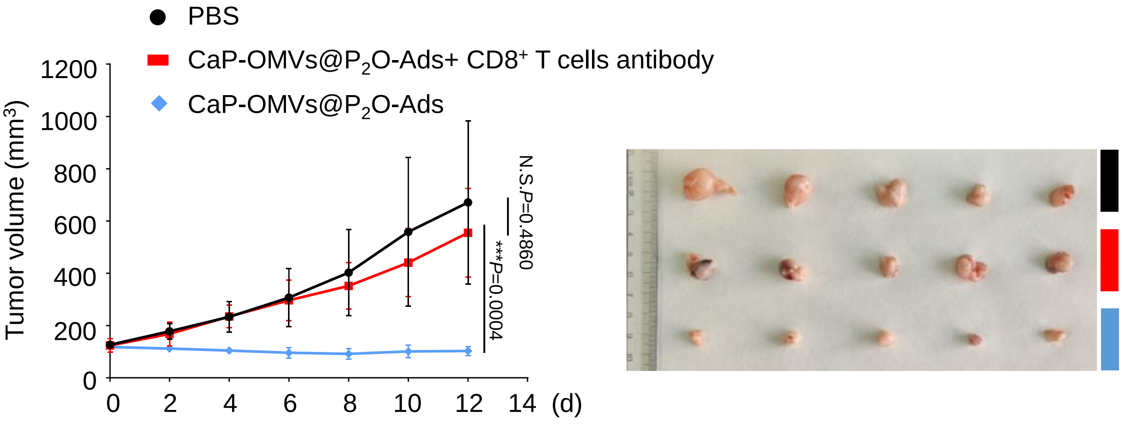


**Supplementary Figure 33.** Images of representative tumors of different treated groups on the 12th day. Data are presented as mean ± SD (n = 5 mice). *$P < 0.05$, ***$P < 0.001$ by two-tailed Student's t-test. Source data are provided as a Source Data file.

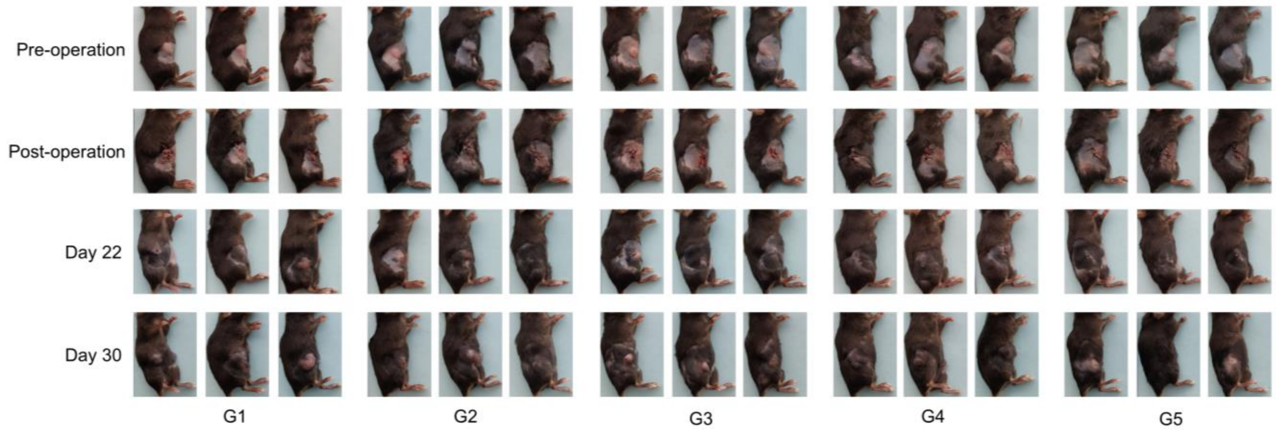


**Supplementary Figure 34.** Representative photos of primary tumor surgery and postoperative recurrence. (G1: PBS, G2: OMVs@$P_2O$-Ads, G3: CaP-OMVs-Ads, G4: Intra-Ads, G5: CaP-OMVs@$P_2O$-Ads).

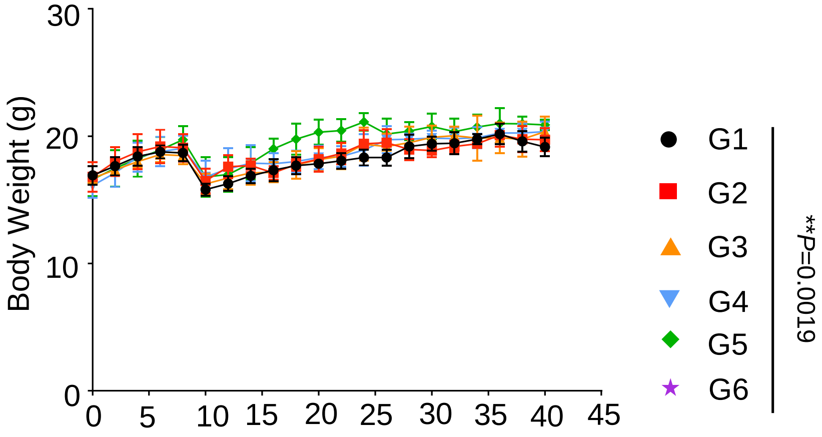


**Supplementary Figure 35.** Body weight changes of TC-1-bearing mice after administration of different formulations. Data are presented as mean ± SD (n = 5 mice). N.S. (No Significance) $P >$ 0.05 by two-tailed Student's t-test. Source data are provided as a Source Data file. (G1: PBS, G2: OMVs@$P_2O$-Ads, G3: CaP-OMVs-Ads, G4: Intra-Ads, G5: CaP-OMVs@$P_2O$-Ads).

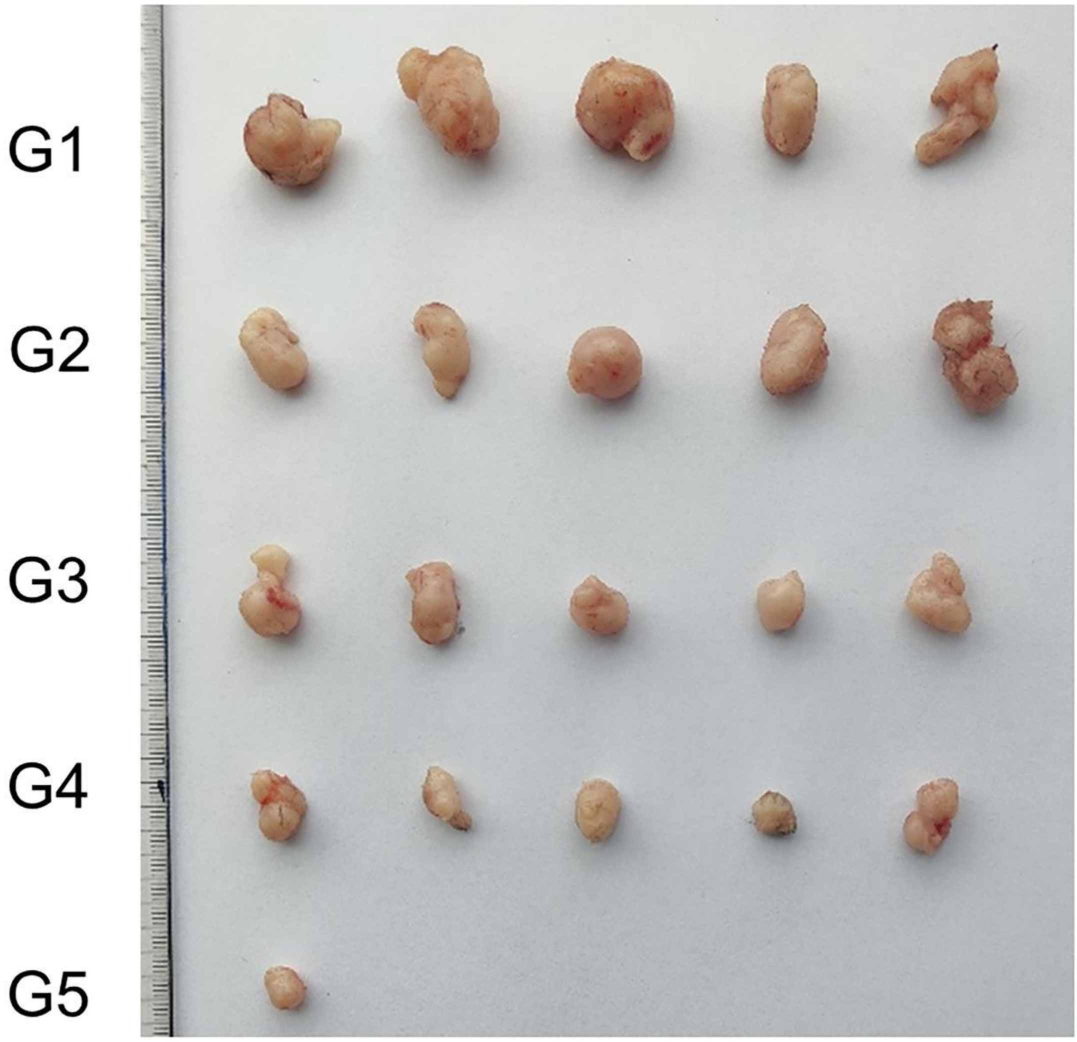


**Supplementary Figure 36.** Images of representative primary tumors after different treatments at 45 days (n=5). (G1: PBS, G2: OMVs@$P_2O$-Ads, G3: CaP-OMVs-Ads, G4: Intra-Ads, G5: CaP-OMVs@$P_2O$-Ads).

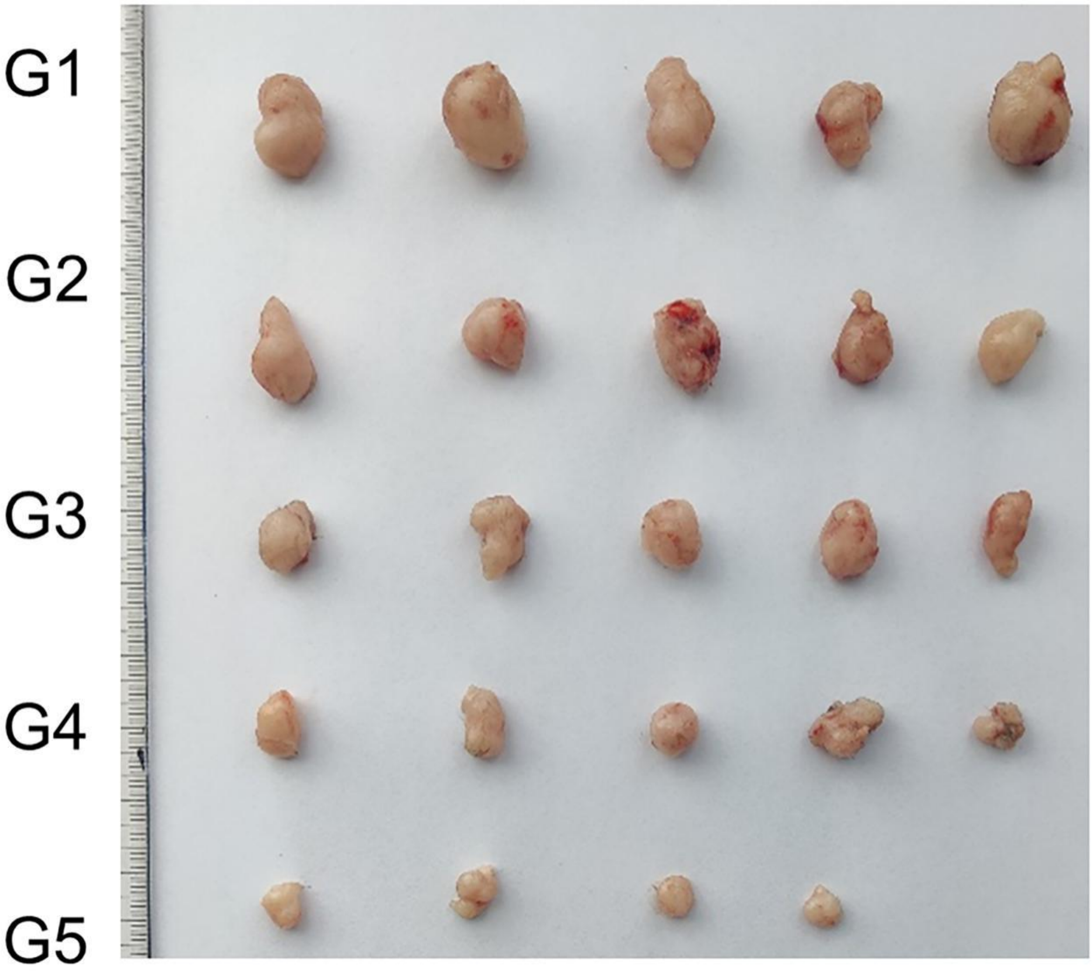


**Supplementary Figure 37.** Images of representative distant tumors after different treatments at 45 days (n=5). (G1: PBS, G2: OMVs@$P_2O$-Ads, G3: CaP-OMVs-Ads, G4: Intra-Ads, G5: CaP-OMVs@$P_2O$-Ads).

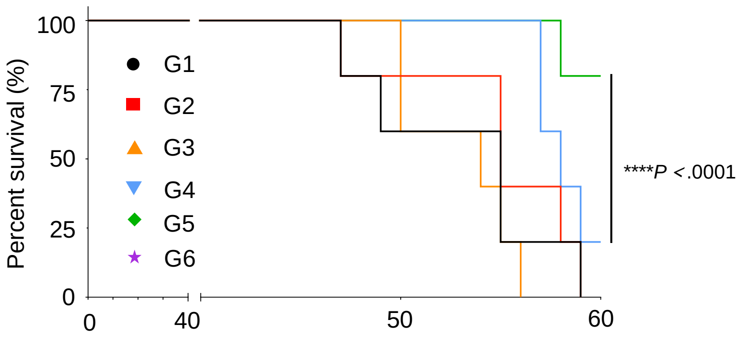


**Supplementary Figure 38.** Survival curves for treated bodies. Data are presented as mean ± SD (n = 5 mice). ****$P$ < 0.001 by two-tailed Student's t-test. Source data are provided as a Source Data file. (G1: PBS, G2: OMVs@$P_2O$-Ads, G3: CaP-OMVs-Ads, G4: Intra-Ads, G5: CaP-OMVs@$P_2O$-Ad)

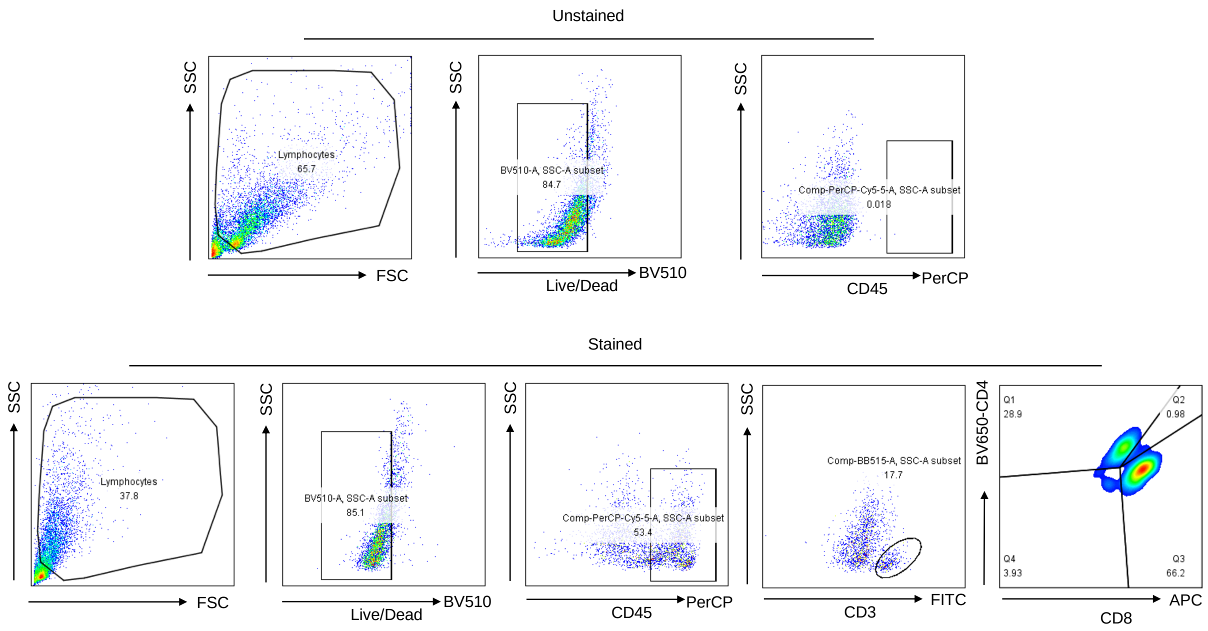


**Supplementary Figure 39.** The gating strategy of $CD8^+$ T cells ($CD45^+CD3^+CD8^+$) in the tumor. (cf. Figure 5d and Supplementary Figures 14 and 24)

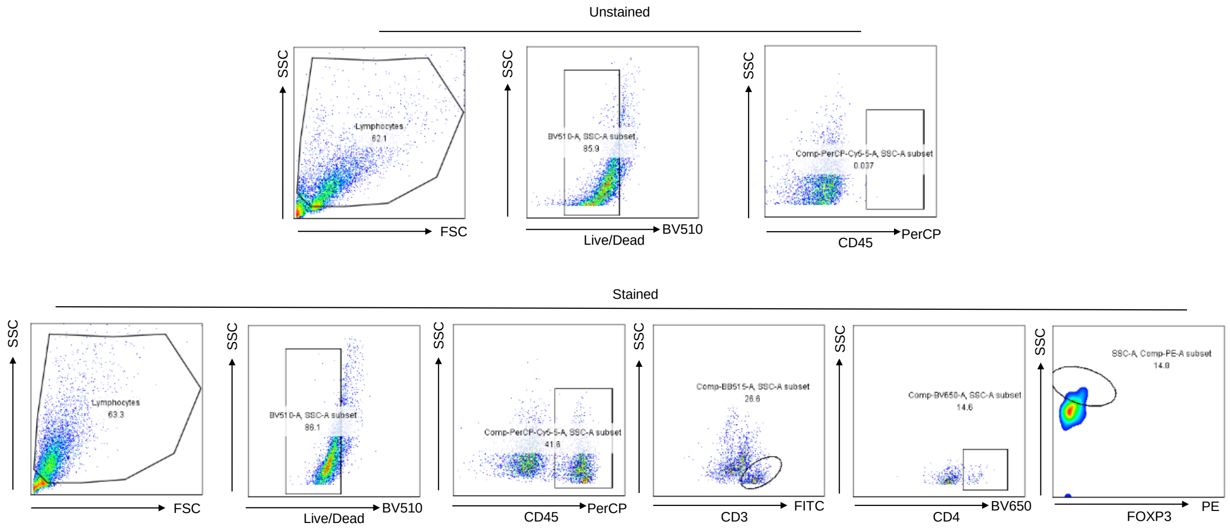


**Supplementary Figure 40.** The gating strategy of Treg cells (CD45$^{+}$CD3$^{+}$CD4$^{+}$FOXP3$^{+}$) in the tumor. (cf. Figure 5e)

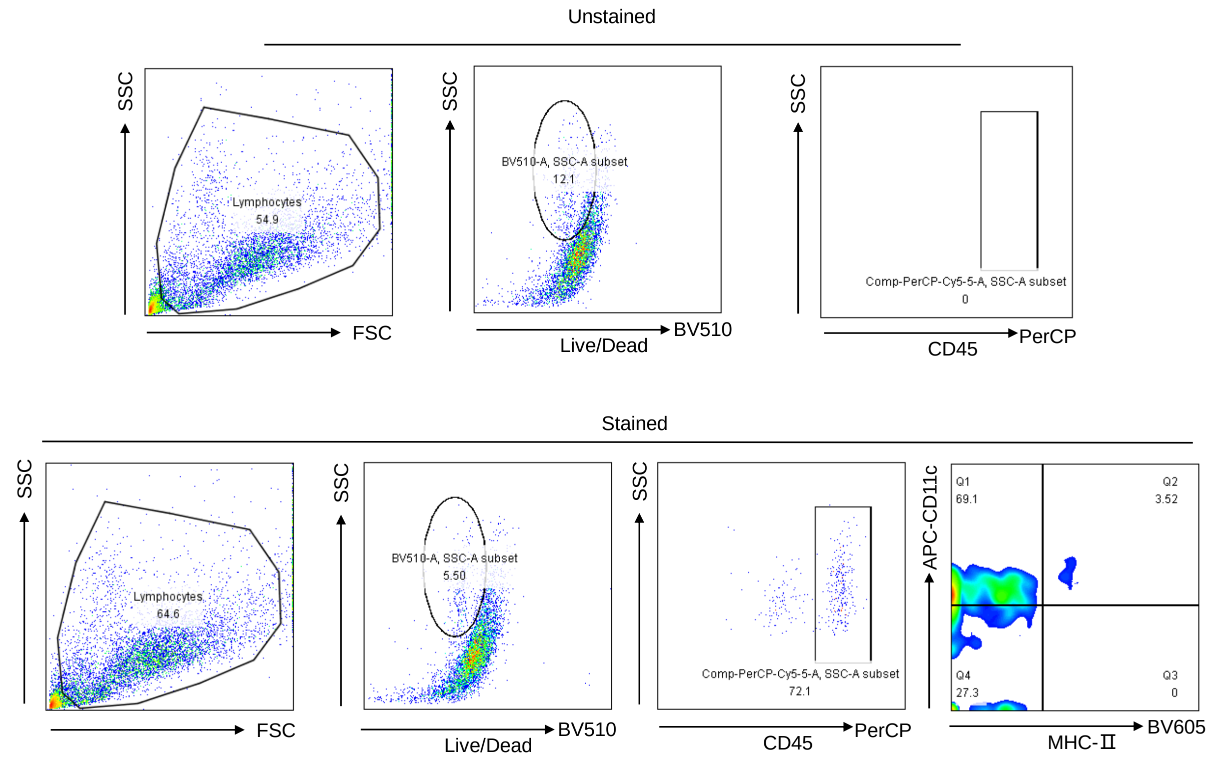


**Supplementary Figure 41.** The gating strategy of MHC-II$^{+}$ DC cells (CD45$^{+}$CD11c$^{+}$MHC-II$^{+}$) in the tumor. (cf. Figure 5f)

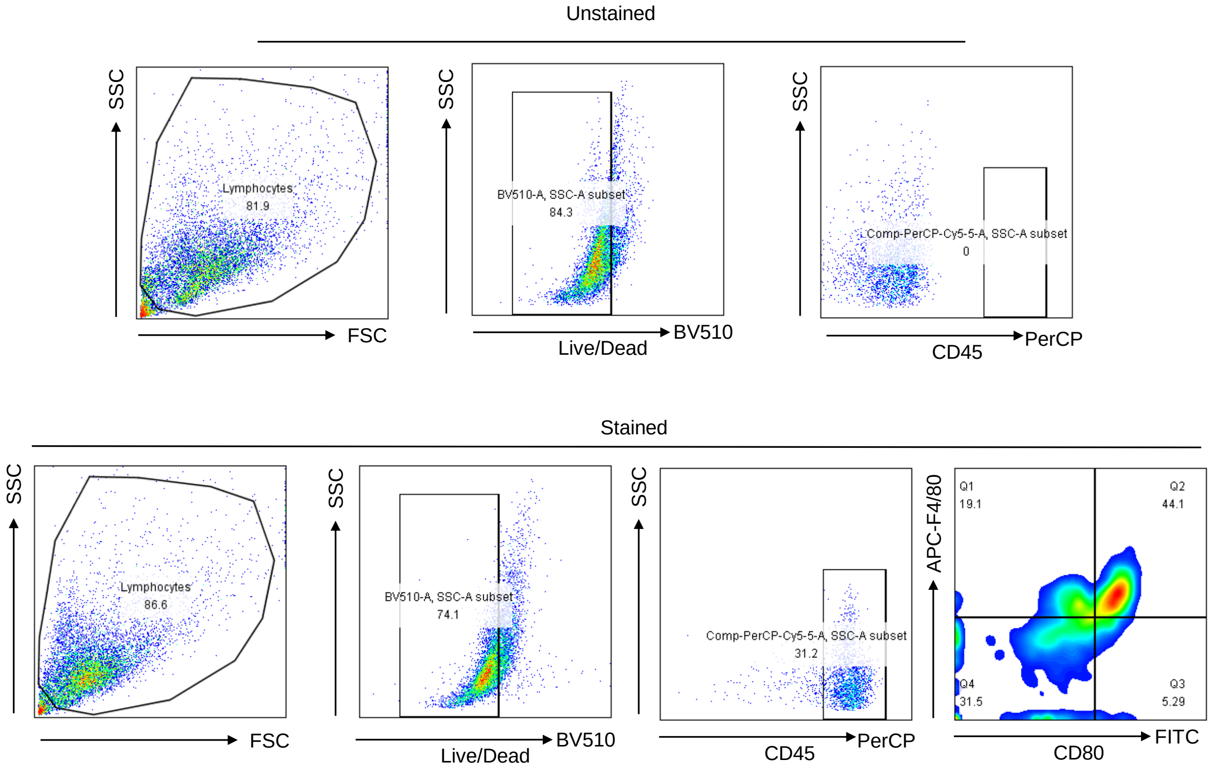


**Supplementary Figure 42.** The gating strategy of M1-like macrophages (CD45$^{+}$F4/80$^{+}$CD80$^{+}$) in tumor. (cf. Supplementary Figure 26)

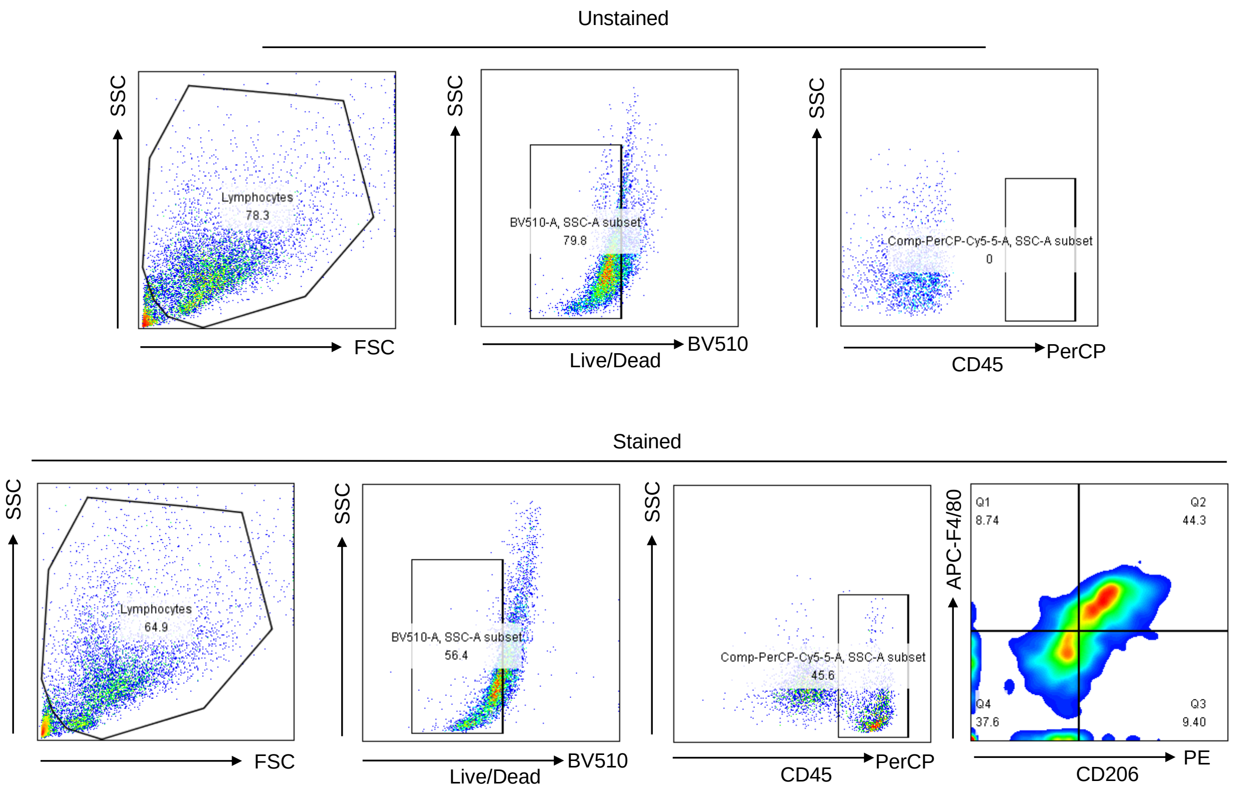


**Supplementary Figure 43.** The gating strategy of M2-like macrophages (CD45$^+$F4/80$^+$CD206$^+$) in tumor. (cf. Supplementary Figure 28)

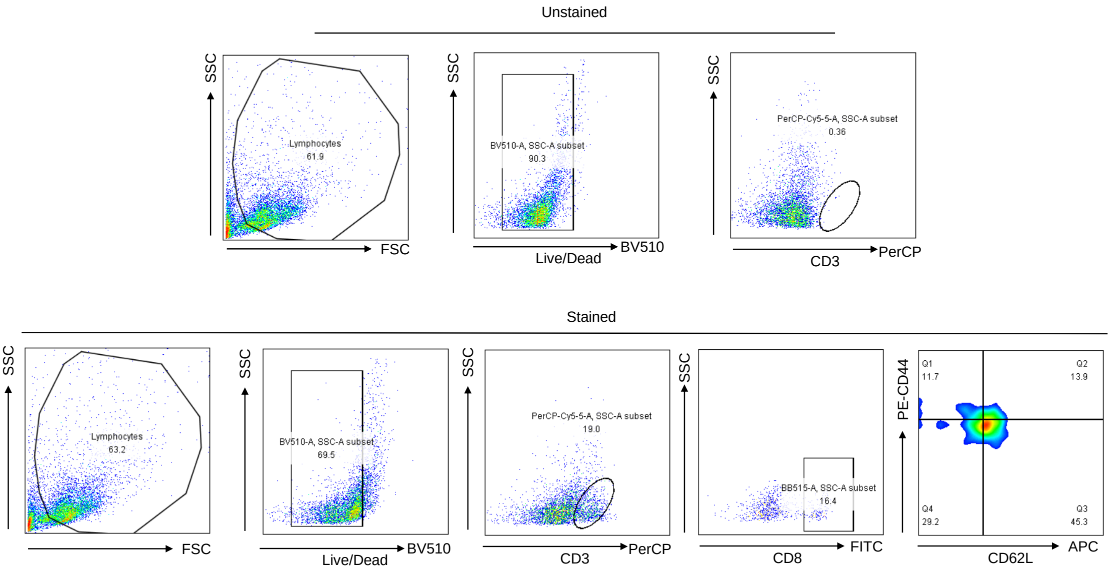


**Supplementary Figure 44.** The gating strategy of effector memory T cells ($CD3^{+}$ $CD8^{+}$ $CD62L^{-}$ $CD44^{+}$) in spleen. (cf. Supplementary Figure 30)

## Supplementary Tables

**Supplementary Table 1. Information on antibodies used in this study**

| Name | Dilution | Validation | Company name | catalog number | Clone number |
|---|---|---|---|---|---|
| LC3B pAb | 1:1000 | WB | ABCLONAL | A5601 | No |
| SQSTM1/p62 | 1:100 | IF | ABCLONAL | A11247 | No |
| LC3B pAb | 1:100 | IF | ABCLONAL | A11282 | No |
| CD8b Antibody | 1:100 | IF | Abmart Inc. | PK12778 | No |
| CD45 Antibody | 1:250 | Flow | Biolegend | 103130 | 30-F11 |
| Fixable Viability Stain 510 | 1:250 | Flow | BD Biosciences | 564406 | No |
| BV650 Rat Anti-Mouse IFN-γ(XMG1.2) | 1:250 | Flow | BD Biosciences | 563854 | XMG1.2 |
| BV605 Rat Anti-Mouse I-A/I-E(M5/114.15.2 ) | 1:250 | Flow | BD Biosciences | 563413 | M5/114.15.2 |
| CD3 Antibody | 1:250 | Flow | Biolegend | 100204 | 17A2 |
| CD8a Antibody | 1:250 | Flow | Biolegend | 100712 | 53-6.7 |
| FOXP3 Antibody | 1:250 | Flow | Biolegend | 126404 | MF-14 |
| CD69 Antibody | 1:250 | Flow | BD Biosciences | 562920 | H1.2F3 |

| | | | | | |
|---|---|---|---|---|---|
| MHC-II Antibody | 1:250 | Flow | BD Biosciences | 563413 | M5/114.15.2 |
| CD11c Antibody | 1:250 | Flow | Biolegend | 117324 | N418 |
| F4/80 Antibody | 1:250 | Flow | Biolegend | 123118 | BM8 |
| CD62L Antibody | 1:250 | Flow | Biolegend | 104428 | MEL-14 |
| CD44 Antibody | 1:250 | Flow | Biolegend | 103008 | IM7 |
| CD80 Antibody | 1:250 | Flow | Biolegend | 104714 | 16-10A1 |
| CD206 Antibody | 1:250 | Flow | Biolegend | 141706 | C068C2 |
| CD4 Antibody | 1:250 | Flow | BD Biosciences | 563232 | GK1.5 |
| CD80 Antibody | 1:250 | Flow | Biolegend | 104706 | 16-10A1 |
| MOUSE CD8a | 5mg | Injection | BIOXCELL | BP0061 | No |
