# Supplementary material for: Engineered bacterial outer membrane vesicles encapsulating oncolytic adenoviruses enhance the efficacy of cancer virotherapy by augmenting tumor cell autophagy
Source: Nat Commun. 2023 May 22;14:2933. doi: 10.1038/s41467-023-38679-z (PMC10203215; doi:10.1038/s41467-023-38679-z)
Supplement: Supplementary file 3 — Reporting Summary [file 41467_2023_38679_MOESM3_ESM.pdf]

nature portfolio

Corresponding author(s): Jin Sun

Last updated by author(s): Apr 13, 2023

Reporting Summary

Nature Portfolio wishes to improve the reproducibility of the work that we publish. This form provides structure for consistency and transparency in reporting. For further information on Nature Portfolio policies, see our Editorial Policies and the Editorial Policy Checklist.

Statistics

For all statistical analyses, confirm that the following items are present in the figure legend, table legend, main text, or Methods section.

n/a	Confirmed	
☐	☒	The exact sample size (*n*) for each experimental group/condition, given as a discrete number and unit of measurement
☐	☒	A statement on whether measurements were taken from distinct samples or whether the same sample was measured repeatedly
☐	☒	The statistical test(s) used AND whether they are one- or two-sided<br>*Only common tests should be described solely by name; describe more complex techniques in the Methods section.*
☐	☒	A description of all covariates tested
☐	☒	A description of any assumptions or corrections, such as tests of normality and adjustment for multiple comparisons
☐	☒	A full description of the statistical parameters including central tendency (e.g. means) or other basic estimates (e.g. regression coefficient) AND variation (e.g. standard deviation) or associated estimates of uncertainty (e.g. confidence intervals)
☐	☒	For null hypothesis testing, the test statistic (e.g. *F*, *t*, *r*) with confidence intervals, effect sizes, degrees of freedom and *P* value noted<br>*Give P values as exact values whenever suitable.*
☒	☐	For Bayesian analysis, information on the choice of priors and Markov chain Monte Carlo settings
☐	☒	For hierarchical and complex designs, identification of the appropriate level for tests and full reporting of outcomes
☒	☐	Estimates of effect sizes (e.g. Cohen's *d*, Pearson's *r*), indicating how they were calculated

Our web collection on statistics for biologists contains articles on many of the points above.

Software and code

Policy information about availability of computer code

Data collection	Malvern: Zetasizer Software 7.01; Microplate reader: SkanIt 2.4.3.37; Confocal laser scanning microscopy: NIS 4.13
Data analysis	GraphPad prism 8.0; Microsoft Office 2019; ImageJ 1.8.0; FlowJo_V10; Trim-galore (version 0.6.7); HISAT2 (version 2.2.1); featureCounts (version 2.0.1); DESeq2 (version 1.32.0); R package clusterProfiler

For manuscripts utilizing custom algorithms or software that are central to the research but not yet described in published literature, software must be made available to editors and reviewers. We strongly encourage code deposition in a community repository (e.g. GitHub). See the Nature Portfolio guidelines for submitting code & software for further information.

Data

Policy information about availability of data

All manuscripts must include a data availability statement. This statement should provide the following information, where applicable:

- Accession codes, unique identifiers, or web links for publicly available datasets
- A description of any restrictions on data availability
- For clinical datasets or third party data, please ensure that the statement adheres to our policy

The next-generation-sequencing data generated by this study have been deposited to GEO database under accession number: GSE225094 (https.ncbi.nlm.nih.gov/geo/query/acc.cgi?acc=GSE225094). All data supporting the findings of this study are available within the Article, Supplementary Information or Source Data file. The source data underlying Figure 2a, 2f, 2h, 3c, 3e, 3f-h, 4a, 4d, 4f-h, 5b, 5g-i, 6b, 6d, Supplementary Figure 4, 5, 8, 9, 10, 12, 13,14, 15, 18, 19, 20, 22, 25, 27, 29, 31, 32, 33, 35and 38 have been deposited in the Figshare database ((https://doi.org/10.6084/m9.figshare.22664044).

Human research participants

Policy information about studies involving human research participants and Sex and Gender in Research.

Reporting on sex and gender	No Human research participants were involved in this manuscript
Population characteristics	No Human research participants were involved in this manuscript
Recruitment	No Human research participants were involved in this manuscript
Ethics oversight	No Human research participants were involved in this manuscript

Note that full information on the approval of the study protocol must also be provided in the manuscript.

Field-specific reporting

Please select the one below that is the best fit for your research. If you are not sure, read the appropriate sections before making your selection.

- [x] Life sciences
- [ ] Behavioural & social sciences
- [ ] Ecological, evolutionary & environmental sciences

For a reference copy of the document with all sections, see nature.com/documents/nr-reporting-summary-flat.pdf

Life sciences study design

All studies must disclose on these points even when the disclosure is negative.

Sample size	The sample size was 3 for in vitro experiments, 4 for in vivo distribution experiments, 5 or 6 for pharmacodynamic experiments, and 3 for immunological studies. We made every effort to ensure that the sample size was adequate.
Data exclusions	No data were excluded from the analyses.
Replication	In this study, the experiment was repeated independently 3 times.
Randomization	Samples were randomly allocated into experimental groups.
Blinding	The investigators were blinded to group allocation during data collection and analysis.

Reporting for specific materials, systems and methods

We require information from authors about some types of materials, experimental systems and methods used in many studies. Here, indicate whether each material, system or method listed is relevant to your study. If you are not sure if a list item applies to your research, read the appropriate section before selecting a response.

Materials & experimental systems

n/a	Involved in the study
[ ]	[x] Antibodies
[ ]	[x] Eukaryotic cell lines
[x]	[ ] Palaeontology and archaeology
[ ]	[x] Animals and other organisms
[x]	[ ] Clinical data
[x]	[ ] Dual use research of concern

Methods

n/a	Involved in the study
[x]	[ ] ChIP-seq
[ ]	[x] Flow cytometry
[x]	[ ] MRI-based neuroimaging

Antibodies

Antibodies used

LC3B pAb (Company: ABCLONAL; cat: A5601; clone: NO); SQSTM1/p62 (Company: ABCLONAL; cat: A11247; clone: NO); LC3B pAb (Company: ABCLONAL; cat: A11282; clone: NO); CD8b Antibody (Company: ABCLONAL; cat: A11247; clone: NO); CD45 Antibody Company: Biolegend; cat: 103130; clone: 30-F11); Fixable Viability Stain 510 (Company: BD Biosciences; cat: 564406; clone: No); BV650 Rat Anti-Mouse IFN-γ(XMG1.2) (Company: BD Biosciences; cat: 563854; clone: XMG1.2) BV605 Rat Anti-Mouse I-A/I-E (M5/114.15.2) (Company: BD Biosciences; cat: 563413; clone: M5/114.15.2); CD3 Antibody (Company: Biolegend; cat: 100204; clone: 17A2); CD8a Antibody (Company: Biolegend; cat: 100712; clone: 53-6.7); FOXP3 Antibody (Company: Biolegend; cat: 126404; clone: MF-14); CD69 Antibody (Company: BD Biosciences; cat: 562920; clone: H1.2F3); MHC-Ⅱ Antibody (Company: BD Biosciences; cat: 563413; clone: M5/114.15.2); CD11c Antibody (Company: Biolegend; cat: 117324; clone: N418); F4/80 Antibody (Company: Biolegend; cat: 123118; clone: BM8); CD62L Antibody (Company: Biolegend; cat: 104428; clone: MEL-14); CD44 Antibody (Company: Biolegend; cat: 103008; clone: IM7); CD80 Antibody (Company: Biolegend; cat: 104714; clone: 16-10A1); CD206 Antibody (Company: Biolegend; cat: 141706; clone: C068C2); CD4 Antibody (Company: BD Biosciences; cat: 563232; clone: GK1.5); CD80 Antibody (Company: Biolegend; cat: 141706; clone: 16-10A1); MOUSE CD8a (Company: BIOXCELL; cat: BP0061; clone: No).

Validation

LC3B pAb (Company: ABCLONAL; cat: A5601; clone: NO):WB; SQSTM1/p62 (Company: Abmart Inc.; cat: PK12778; clone: NO): IF; LC3B pAb (Company: ABCLONAL; cat: A11282; clone: NO): IF; CD8b Antibody (Company: ABCLONAL; cat: A11247; clone: NO): IF; CD45 Antibody Company: Biolegend; cat: 103130; clone: 30-F11): Flow; CD45 Antibody Company: Biolegend; cat: 103130; clone: 30-F11); Fixable Viability Stain 510 (Company: BD Biosciences; cat: 564406; clone: No); BV650 Rat Anti-Mouse IFN-γ(XMG1.2) (Company: BD Biosciences; cat: 563854; clone: XMG1.2) BV605 Rat Anti-Mouse I-A/I-E(M5/114.15.2) (Company: BD Biosciences; cat: 563413; clone: M5/114.15.2); CD3 Antibody (Company: Biolegend; cat: 100204; clone: 17A2); CD8a Antibody (Company: Biolegend; cat: 100712; clone: 53-6.7); FOXP3 Antibody (Company: Biolegend; cat: 126404; clone: MF-14); CD69 Antibody (Company: BD Biosciences; cat: 562920; clone: H1.2F3); MHC-Ⅱ Antibody (Company: BD Biosciences; cat: 563413; clone: M5/114.15.2); CD11c Antibody (Company: Biolegend; cat: 117324; clone: N418); F4/80 Antibody (Company: Biolegend; cat: 123118; clone: BM8); CD62L Antibody (Company: Biolegend; cat: 104428; clone: MEL-14); CD44 Antibody (Company: Biolegend; cat: 103008; clone: IM7); CD80 Antibody (Company: Biolegend; cat: 104714; clone: 16-10A1); CD206 Antibody (Company: Biolegend; cat: 141706; clone: C068C2); CD4 Antibody (Company: BD Biosciences; cat: 563232; clone: GK1.5); CD80 Antibody (Company: Biolegend; cat: 141706; clone: 16-10A1): flow; MOUSE CD8a (Company: BIOXCELL; cat: BP0061; clone: No): Injection.

Eukaryotic cell lines

Policy information about cell lines and Sex and Gender in Research

Cell line source(s)

HCT116 cell line, the modified non-small cell lung (TC-1) cancer cell line expressing the human CD46 receptor and TC-1-hCD46-luc cell line were the kindly gift from the Sino-British Research Centre of Zhengzhou University.

Authentication

As TC-1-hCD46 cell was murine, HCT116 cells was human, Cell lines validation using short tandem repeat (STR) markers were performed by Genetic Testing Biotechnology Corporation (Suzhou, China). In detail, eighteen STR loci were amplified using multiplex PCR. One additional marker (Human TH01) was used to screen for the presence of human species. The cell line sample was processed using the ABI Prism 3130 XL Genetic Analyzer. Data were analyzed using Gene Mapper ID 3.2 software (Applied Biosystems). Appropriate positive and negative controls were run and confirmed for each sample submitted. The HCT116 cell line, TC-1-hCD46 cell line and TC-1-hCD46-luc cell line were maintained in Dulbecco's modified Egle's medium (DMEM) with 10% FCS.

Mycoplasma contamination

All cell lines tested negative for mycoplasma contamination.

Commonly misidentified lines
(See ICLAC register)

No commonly misidentified cells lines were used in the study.

Animals and other research organisms

Policy information about studies involving animals; ARRIVE guidelines recommended for reporting animal research, and Sex and Gender in Research

Laboratory animals

C57BL/6JOlaHsd mice, 8-10 weeks old

Wild animals

The study did not involve wild animals.

Reporting on sex

Female

Field-collected samples	The study did not involve samples collected from the field.
Ethics oversight	Our research complies with all relevant ethical regulations. All the animal protocols were performed in line with the Guidelines for the Care and Use of Laboratory Animals and approved by the Institutional Animal Ethical Care Committee (IAEC) of Shenyang Pharmaceutical University.

Note that full information on the approval of the study protocol must also be provided in the manuscript.

Flow Cytometry

Plots

Confirm that:

- [x] The axis labels state the marker and fluorochrome used (e.g. CD4-FITC).
- [x] The axis scales are clearly visible. Include numbers along axes only for bottom left plot of group (a 'group' is an analysis of identical markers).
- [x] All plots are contour plots with outliers or pseudocolor plots.
- [x] A numerical value for number of cells or percentage (with statistics) is provided.

Methodology

Sample preparation	Peripheral spleens and tumor tissues were extracted from TC-1-hCD46 bearing C57 mice. Spleen and tumor tissue were first used to create a single-cell suspension.
Instrument	Flow cytometer (BD FACS Celesta)
Software	FlowJo_V10
Cell population abundance	Mouse tumor tissue and spleen cell suspensions were obtained by lymphocyte isolation.
Gating strategy	By labeling the corresponding cells with the corresponding antibody with fluorescence, the purpose of screening is achieved (e.g. CD4-FITC). We determined the positive and negative cell populations by setting blank control groups. The specific gating strategy has been shown in the Supplementary Information (Supplementary Figures 42-47). The reference is provided here (https://doi.org/10.1038/s41551-022-00886-2).

- [x] Tick this box to confirm that a figure exemplifying the gating strategy is provided in the Supplementary Information.
